# Molecular dissection of the genetic architecture of phenology underlying *Lupinus hispanicus* early flowering and adaptation to winter- or spring sowing

**DOI:** 10.1038/s41598-025-00096-1

**Published:** 2025-05-02

**Authors:** Wojciech Bielski, Anna Surma, Jolanta Belter, Bartosz Kozak, Michał Książkiewicz, Sandra Rychel-Bielska

**Affiliations:** 1 Department of Gene Structure and Function, Institute of Plant Genetics, Polish Academy of Sciences, Poznan, 60-479 Poland; 2 Department of Genetics and Plant Breeding, Poznań University of Life Sciences, Dojazd 11, Poznan, 60- 632 Poland; 3 Department of Genetics, Plant Breeding and Seed Production, Wroclaw University of Environmental and Life Sciences, Wroclaw, 50-363 Poland

**Keywords:** *Lupinus hispanicus*, GWAS, Flowering induction, Vernalization, Legumes, Legume crop, Plant breeding, Plant development, Plant genetics, Plant molecular biology, Agricultural genetics, Genetic markers, Genomics, Plant breeding

## Abstract

Spanish lupin, *Lupinus hispanicus* Boiss. et Reut. is an untapped grain legume species characterized by moderate frost resistance, tolerance to poor soil and waterlogging, high yield stability, and remarkable seed protein content. It has been recognized as a good candidate for domestication to broaden the palette of crop diversity. One of the key characteristics that need to be precisely addressed during domestication is the vernalization responsiveness of flowering, which is advantageous in autumn sowing due to improved frost tolerance, whilst undesired in spring sowing as it delays flowering. Ahead of *L. hispanicus* breeding, in the present work, we aimed to recognize existing phenotypic variability of flowering time and vernalization response and to evaluate the genetic architecture of early and late phenology by DArT-seq genotyping and genome-wide association study (GWAS) in world germplasm collection of the species. Controlled environment phenotyping revealed high variability of flowering time and vernalization responsiveness and significant correlations with population structure. DArT-seq genotyping yielded 23 728 highly polymorphic markers distributed extensively across all 26 chromosomes. GWAS identified a number of markers significantly associated with flowering time with or without pre-sowing vernalization, including those overlapping with the two major quantitative trait loci reported previously for white lupin species. Microsynteny-based analysis of the genetic content of *L. hispanicus* genome regions carrying significantly associated markers highlighted several candidate genes from photoperiodic and vernalization pathways. To summarize, the present study identified germplasm resources for autumn- and spring-sown cultivation of *L. hispanicus* and provided tools for marker-assisted selection towards required flowering phenology.

**Supplementary Information:**

The online version contains supplementary material available at 10.1038/s41598-025-00096-1.

## Introduction

Domesticated lupin species are among the most valuable crops due to their natural adaptation to wide range of environments and appreciated grain chemical composition. Their seeds contain high concentrations of essential amino acids and proteins, and they produce stable amounts oils with exceptional nutritional characteristics, including high levels of unsaturated fatty acids and tocopherols. These are important in the human diet and are considered as one of the best alternatives to soybean protein^1,2^. Additionally, due to the rising global average temperature and changes in precipitation patterns, there is a growing need for plants that can thrive in dry, sandy soils^3^. This is a typical characteristic of numerous lupin species that differentiates them from many other grain legume crops^4,5,6,7^. Nowadays, lupins are gaining in popularity as high-protein grain crops in many countries^8,9,10^.

*Lupinus hispanicus* Boiss. et Reut., Spanish lupin, is characterized by yield stability, waterlogging, poor soil tolerance, and frost resistance^11^. Historically, it has been cultivated experimentally^12,13^. Its high productivity and protein content up to 42%, make it a promising alternative leguminous crop for low-input cultivation^14,15^. It could also contribute to solving local problems of summer forage scarcity by its use as a self-seeding pasture on the premise that the alkaloid content will be reduced^16,17^. The *L. hispanicus* is also a valuable genetic resource for introducing beneficial traits into other, closely related lupin species^18^. Indeed, the natural crosses between the subspecies *L. hispanicus* subsp. bicolor and yellow lupin (*L. luteus*) were observed in Spain, and the first interspecific hybrids were created over 25 years ago^19^.

Now, *L. hispanicus* is being considered as a potential new source of plant protein for modern breeding programs^14^. Nevertheless, domestication of such an untapped wild plant species will require evaluation of existing germplasm diversity panel for many agronomic traits, which include, among others, the length of period from sowing to flowering and its responsiveness to vernalization, aiming at identification of genotypes with desired phenology. Moreover, the ongoing climate change increases the risk of severe weather events, such as prolonged droughts and heatwave events, that may severely affect regions where lupins and other grain legumes are cultivated^20,21^. One of the natural strategies to mitigate risk of yield loss in natural lupin populations is drought escape by early phenology, observed in narrow-leafed (*L. angustifolius*), white (*L. albus*) and yellow lupin germplasm and driven by deletions in the promoter region of floral pathway integrator gene, *Flowering locus T*^22,23,24,25^. It is anticipated that precise adaptation of sowing dates, growing periods and cultivars maturity to local conditions would reduce negative impacts of climate change on crop performance, providing even some yield increase due to the positive CO_2_ fertilization effect^26^. Therefore, one of the biggest challenges for breeding new species is fine-tuning of flowering and maturity dates to given agro-climatic conditions. It is worth noting that the recent climate change already resulted in yield instability of winter crops requiring prolonged chilling period to maintain the process of vernalization and promote transition from vegetative phase to flowering^27,28,29,30^.

Vernalization responsiveness is one of the key characteristics of many lupin species that needs to be precisely addressed in breeding programs to improve resilience to changing climate^31^. This trait is crucial for many crops, including white lupin, pea, and faba bean, because it provides adaptation to autumn sowing in areas with mild winters with some occurrences of freezing temperatures^32,33^. In these areas, appropriate fulfillment of moderate or high vernalization requirement not only prevents flowering before the winter but also prevents from frost damage due to a positive correlation between vernalization responsiveness and frost tolerance during winter^34^. Therefore, the high vernalization requirement of some lupin accessions is one of their most important advantages for autumn or winter sowing in warmer agroclimate, where long juvenile phase is beneficial^35,36,37^. However, this trait is undesirable for spring sowing, because it may considerably delay flowering when vernalization requirement is not fulfilled due to temperatures above vernalization threshold. Moreover, excessive heat may even erase epigenetic marks and result in late flowering phenotype despite correctly addressed vernalization requirements^38^.

To summarize, uncovering the mechanism of flowering induction and vernalization response in the new promising candidate crop species is essential for its future breeding. Genetic determinants and their molecular mechanisms underlying observed phenotypic variation should be identified to facilitate reselection of germplasm with desired phenology in the obtained progeny. Several approaches of reduced genome representation sequencing have been successfully implemented in lupins, including massive analysis of cDNA ends (MACE), restriction-site associated DNA sequencing (RAD-seq), genotyping-by-sequencing (GBS) and Diversity Arrays Technology sequencing (DArT-seq)^39,40,41,42,43,44,45^. With recent advances in high-throughput genotyping technologies, genome-wide association studies (GWAS) have become increasingly powerful in detection of sequence polymorphisms associated with various traits, including flowering time and vernalization responsiveness^4,23,46,47,48^. Finally, the pre-publication release of the chromosome-level genome assembly of a closely related lupin species, *L. luteus*, (GenBank BioProject PRJEB74252, assembly GCA_964019355.1), enabled searching for candidate genes in *L. hispanicus* by shared synteny^49^.

Therefore, in the present study we aimed to characterize the set of *L. hispanicus* lines by reduced genome representation sequencing, to determine their flowering time and vernalization responsiveness, and to associate phenotypic traits with the underlying genetic background by GWAS. The germplasm diversity panel carrying 173 *L. hispanicus* genotypes derived from five seedbanks (located in Poland, Germany, Spain, USA and Australia) was phenotyped in greenhouse for plant phenology under ambient long-day photoperiod with and without pre-sowing vernalization treatment. In parallel, the same set of lines was subjected to DArT-seq genotyping, followed by population structure analysis and GWAS exploiting a closely related *L. luteus* genome as a reference (10.21203/rs.3.rs-4171664/v1).

## Results

### *Lupinus hispanicus* diversity panel revealed high variability in plant phenology and vernalization requirements

To evaluate *L. hispanicus* phenology, phenotyping of flowering and maturity was performed in greenhouse in years 2022 and 2023 encompassing at least three biological replicates. Detailed information regarding the results, including the number of days from sowing to (a) floral bud emergence, (b) the start of flowering, and (c) pod maturity, as well as growing degree days (GDD) and daylight hours related to the traits mentioned above, can be found in Supplementary Table S1. Below, we provide aggregate statistics for the presented dataset.

Average values per line were calculated, revealing that the number of days from sowing to the appearance of the first floral bud on the non-vernalized plants ranged from 65.0 to 150.0 days in 2022 and from 84.1 to 150.0 days in 2023. To facilitate planned GWAS analysis, lines which remained in good condition until the end of experiment but did not develop any floral bud after 150 days from sowing due to strong vernalization requirements were assigned with values of 150 days for the floral bud emergence, 160 days for the start of flowering and missing data for maturity. The vernalization process accelerated the floral bud emergence in the least responsive accessions by 13.0 days in 2022 and 16.8 days in 2023, whereas in the most responsive genotypes by 94.0 days in 2022 and 89.3 days in 2023. The appearance of the first fully colored petal (start of flowering) on the non-vernalized plants was observed after 79.3 to 160.0 days in 2022 and after 93.6 to 163.0 days in 2023. Vernalization responsiveness manifested as acceleration of flowering by 15.2 to 93.0 days in 2022 and 16.9 to 90.3 days in 2023. An average number of days from the floral bud emergence to the start of flowering for non-vernalized plants ranged from 7.0 to 21.0 days in 2022 and from 6.7 to 25.0 days in 2023. These values were similar also for vernalized plants, ranging from 6.0 to 17.3 days and from 5.0 to 13.8 days, respectively.

The number of days from sowing to pod maturity on the main stem ranged from 84.3 to 151.0 days in 2022 and from 98.1 to 136.0 days in 2023. The vernalization process reduced these values by 14.5 to 76.3 days in 2022 and by 14.1 to 51.4 days in 2023. It should be noted here, that due to the lack of the floral bud emergence on some non-vernalized plants until the end of experiment, we cannot provide exact values for vernalization-based acceleration of flowering and maturity for the most responsive accessions.

Despite some thermal differences between the two seasons of greenhouse experiments, the earliest genotypes in 2022 were also very early flowering in 2023, and a similar consistency was found for very late genotypes, highly responsive to vernalization. Consistent flowering time patterns were observed for most of the genotypes, with a few exceptions. The most remarkable differences were observed for lines HIS129, HIS142, HIS062 and HIS108, Namely, lines HIS129 and HIS142 in the year 2022 flowered very early, after 79.3 and 83.5 days from sowing (1st percentile), whereas in the next year they flowered over 42 and 40 days later, reaching 64th and 68th percentile in the analyzed set of lines. Oppositely, accessions HIS062 and HIS108 flowered about 36–39 days earlier in 2023, reaching 56th and 67th percentile, than in 2022 (81st percentile).

**Fig. 1:**
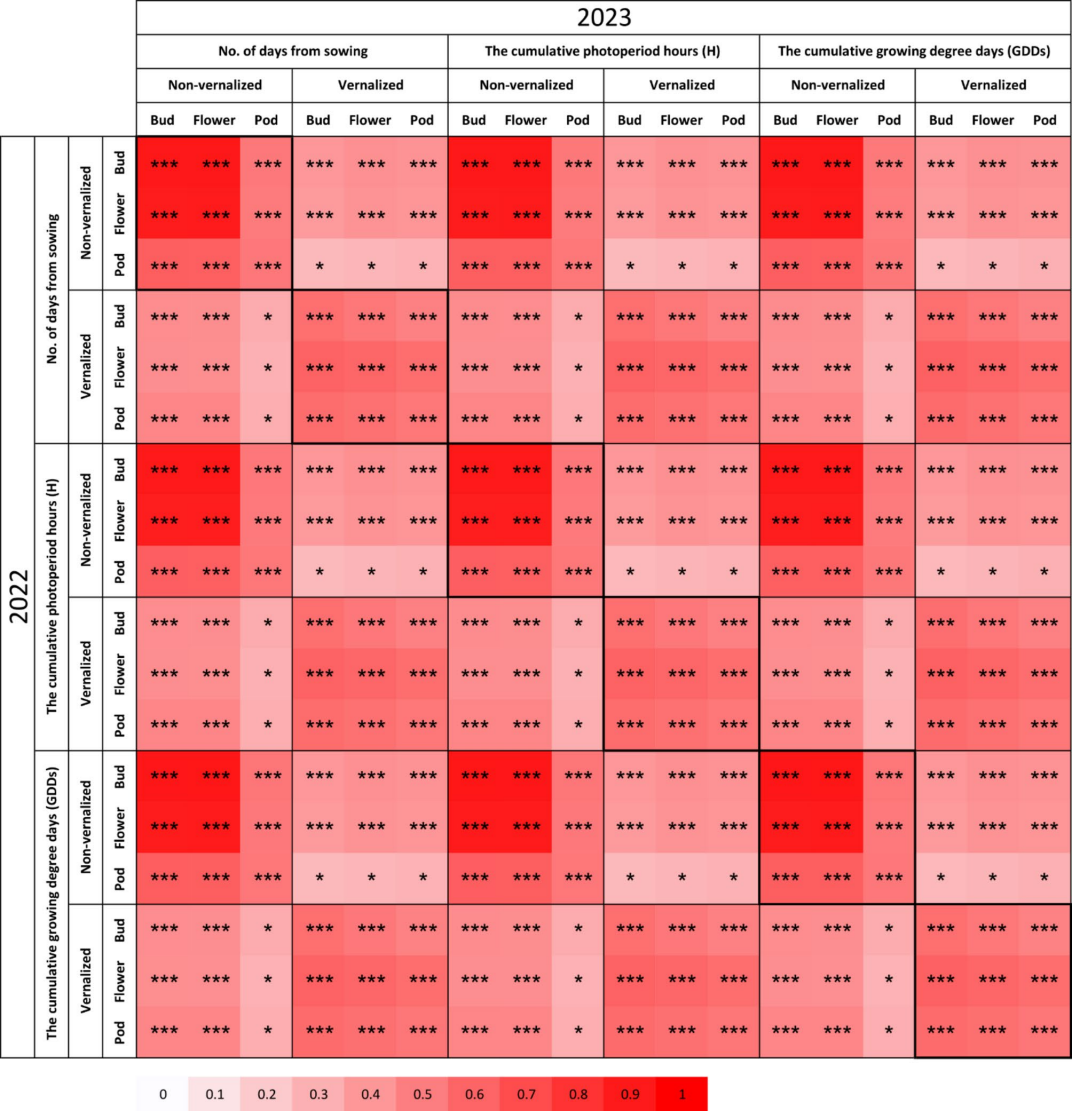
Correlation heatmap reporting Pearson correlation coefficients between years for each trait. Observations were performed during 2022 and 2023 growing seasons in a greenhouse at the Institute of Plant Genetics, Polish Academy of Sciences, Poznań, Poland (52°26′ N 16°54′ E). The bar below the heatmap indicates the color legend of correlation coefficients (from 0 to 1). Asterisk (*) indicates significant correlations in the following scheme: ***, *p* < 0.00001; **, 0.00001 ≤ *p* < 0.0001; *, 0.0001 ≤ *p* ≤ 0.05.

The average standard deviation of the number of days to bud emergence and start of flowering for non-vernalized plants within accessions reached 3.5 and 4.0 days in 2022 as well as 3.0 and 3.7 days in 2023. For vernalized plants, the corresponding values were 1.8 and 2.4 days in 2022, followed by 1.7 and 2.2 days in 2023. The correlation coefficients for the number of days to the floral bud emergence, start of flowering and maturity between years for non-vernalized plants were 0.906, 0.893 and 0.525, whereas for vernalized plants reached 0.562, 0.596 and 0.530 (Fig. 1). Those correlation coefficients were statistically significant with P-values below 1 × 10^− 9^. Data on the number of days from sowing to observed phenological phases (floral bud emergence, start of flowering and pod maturity) were provided in Supplementary Table S1. Generalized broad-sense heritability formula, which accounts for both additive and non-additive genetic variances, yielded values between 0.47 and 0.50 for non-vernalized plants and 0.48–0.49 for the vernalized ones. Calculations were conducted on datasets stratified by year to address potential temporal variability, ensuring results were reflective of annual differences. Nevertheless, differences in heritability between years were negligible, as the mean heritability value for all traits was 0.49 in both years.

Phenotypic observations were supplemented with calculation of the cumulative photoperiod hours and the cumulative growing degree days (GDDs) from sowing to reaching particular phenological phase. Thus, for non-vernalized plants in 2022, the cumulative photoperiod hours from sowing to the floral bud emergence, start of flowering and maturity varied from 886 to 2273, from 1115 to 2422, and from 1197 to 2288 h, respectively. For vernalized plants, the corresponding values were from 662 to 1247, from 793 to 1498, and from 891 to 1532 h. This trait was highly correlated (as expected) with the number of days from sowing to the floral bud emergence, start of flowering and maturity in both years (r-value above 0.999).

The cumulative number of GDDs from sowing to the floral bud emergence, start of flowering and maturity for non-vernalized plants varied in 2022 from 1137.7 to 2949.8, from 1411.6 to 3177.0, and from 1500.9 to 2970.7, respectively. For vernalized plants, the corresponding values were from 860.2 to 1562.0, from 1018.0 to 1882.0, and from 1143.7 to 1924.4. Consequently, in 2022, vernalization accelerated the floral bud emergence from 248.8 to 1988.9 GDDs, flowering from 285 to 2002.4 GDDs and maturity from 272.3 to 1644.8 GDDs. In 2023, these values ranged from 339.7 to 1862.2 GDDs for the floral bud emergence, from 350.1 to 1937.6 GDDs for flowering and from 303.0 to 1122.8 GDDs for maturity. This trait was also highly correlated with the number of days from sowing to the floral bud emergence, start of flowering and maturity in both experiments (r-value above 0.999). Data on the number of cumulative growing degree days (GDDs) and photoperiod hours (H) from sowing until reaching studied phenological phases are provided in Supplementary Table S1.

###  DArT-seq genotyping of ***L. hispanicus*** diversity panel provided 23,728 high quality markers distributed extensively across all 26 pseudochromosomes

Lupin DArT-seq 1.0 protocol yielded a total number of 125 218 SNP markers and 177 026 presence/absence variants (PAVs, SilicoDArTs), from which ~ 107 000 SNPs and 124 000 PAVs were monomorphic). After filtering by reproducibility rate, missing data, heterozygosity and minor allele frequency (MAF) a total of 5959 SNP and 17 769 PAV markers were retained for population structure analysis and GWAS (Supplementary Table S2). The total number of 13359 PAV and 4352 SNP markers were successfully aligned to *L. luteus* genome assembly, whereas 4410 PAV and 1607 SNP markers remained unlinked. The number of aligned markers per chromosome ranged from 376 on chromosome YL-08 to 965 on chromosome YL-09. The average marker density ranged from 9.56 markers/Mbp (chromosome YL-08) to 25.93 markers/Mbp (chromosome YL-25), with mean value of 18.79 markers/Mbp. Ten chromosomes (YL-25, YL-09, YL-18, YL-26, YL-23, YL-22, YL-12, YL-21, YL-10 and YL-13) reached density more than 20 markers/Mbp. There mean gap between markers was 53.8 kbp, however, 98 gaps were larger than 1 Mbp. Three largest gaps between markers were located on chromosomes YL-02 (4.8 Mbp), YL-07 (4.4 Mbp) and YL-08 (4.4 Mbp) (Supplementary Table S3). After missing data imputation, the mean heterozygosity of markers was 3.97%, ranging from 0.00 to 21.97%, with 17 372 markers showing heterozygosity below 5% and 53 markers above 15%. The heterozygosity of genotypes ranged from 0.31 to 27.48%. Within the analyzed germplasm panel, 137 genotypes had heterozygosity below 5%, whereas 7 above 15% (HIS_122, HIS_024, HIS_007, HIS_123, HIS_116, HIS_064 and HIS_226).

### Plant phenology of *L. hispanicus* diversity panel was significantly correlated with population structure

Analysis of the cross-entropy graph for clustering within the range from K3 to K15 revealed a rapid loss of cross-entropy from K = 3 until a minimum located at K = 6, followed by immediate cross-entropy increase continued until the highest analyzed K-value (Supplementary Figure S1). To validate the optimal K-value selection we performed principal component analysis. Visualization of principal component clusters (PC1 vs. PC2 and PC2 vs. PC3) was provided in Supplementary Figure S2.

**Fig. 2:**
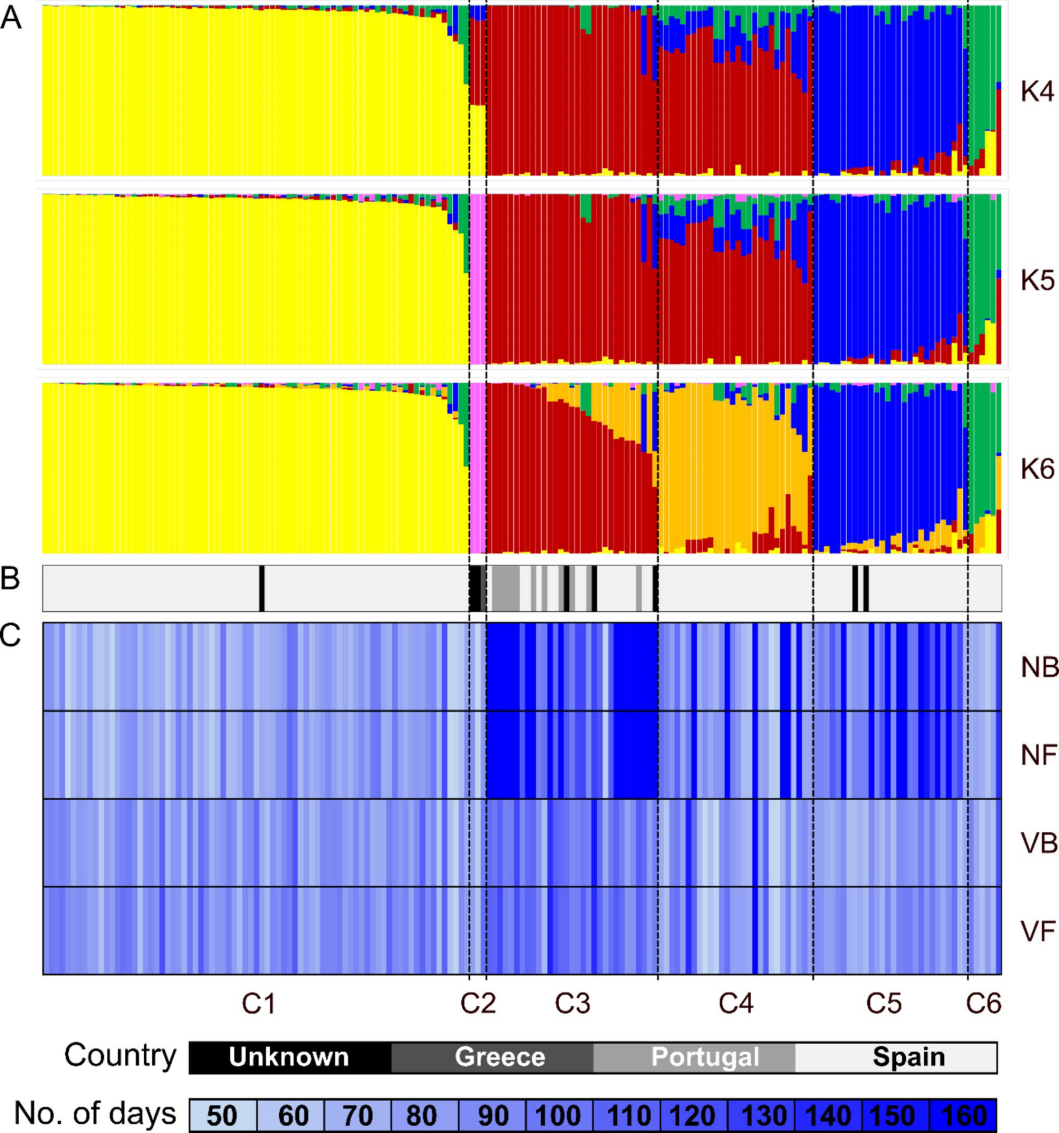
Visualization of population structure analysis of 173 *Lupinus hispanicus* accessions. The panels show (**A**) STRUCTURE diagrams under different K-values (K4, K5 and K6), (**B**) country of origin and (**C**) mean total number of days from sowing to the floral bud emergence and start of flowering for non-vernalized (NB and NF) and vernalized (VB and VF) plants. In total, 5959 SNP and 17,769 PAV markers were used for population structure analysis. Phenotypic observations were performed during 2022 and 2023 growing seasons in a greenhouse at the Institute of Plant Genetics, Polish Academy of Sciences, Poznań, Poland (52°26′ N 16°54′ E). The deeper the shade of blue on the NB, NF, VB and VF panels, the later the phenotype according to the scale provided below the panels.

**Fig. 3:**
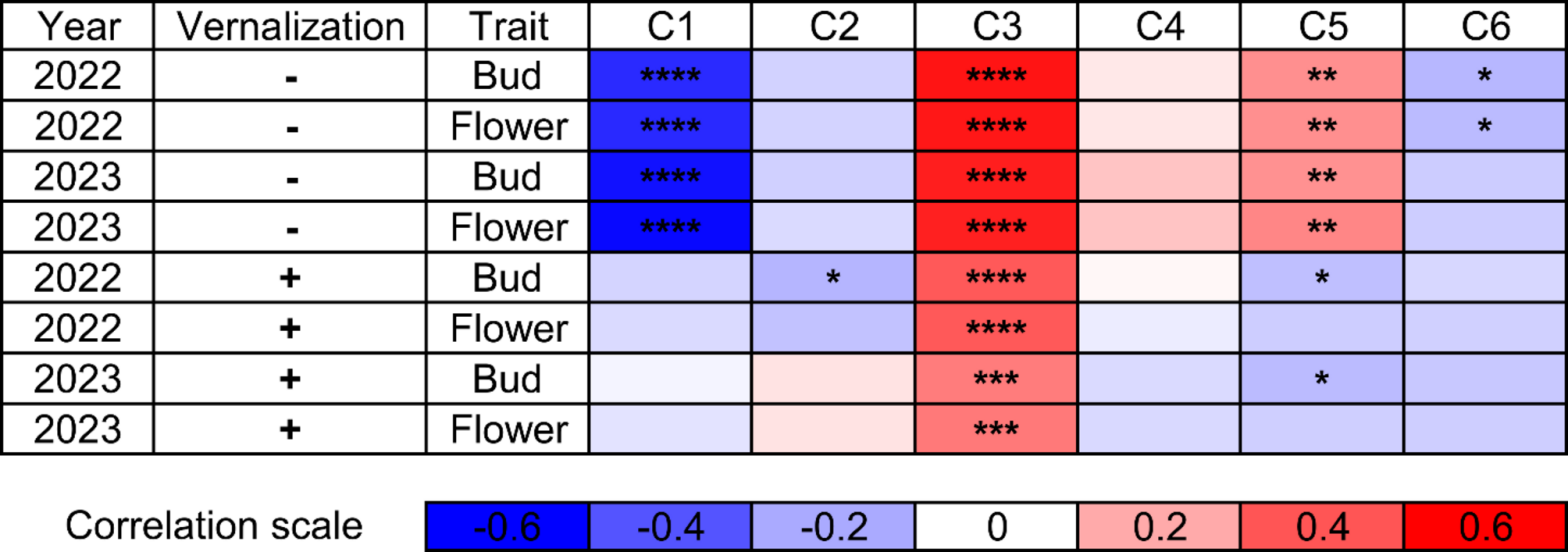
The Heatmap reporting correlation coefficient values and p-values calculated for assignment to 6 clusters and 8 combinations of trait, year and vernalization. Asterisk (*) indicates significant correlations in the following scheme: ****, *p* < 0.00001; ***, 0.00001 ≤ *p* < 0.0001; **, 0.0001 ≤ *p* ≤ 0.001; *, *p* < 0.05.; “ “, non-significant.

K-values within the range of 4–6 were screened for population structure analysis (Supplementary Table S4). Since the first tested K-value (K = 4), grouping started to follow differences in flowering time, especially for non-vernalized plants. As permutation test provides assignment to particular clusters in the continuous range from 0 to 1, we were able to calculate correlation coefficients between observed plant phenology and cluster assignment values. At K = 6 value, selected as the most representative according to the cross-entropy criterion, assignment to three clusters (C1, C3 and C5) revealed significant correlations with phenotypic observations (Figs. 2 and 3). Thus, the largest cluster, C1 carrying 77 accessions, was significantly correlated with earliness of non-vernalized plants, demonstrated by r-values − 0.54 and − 0.55 for the number of days from sowing to the floral bud emergence and start of flowering (p-values 1.8 × 10^–14^ and 2.6 × 10^–15^). The second largest cluster, C3 carrying 31 accessions, was significantly correlated with late phenology in both non-vernalized and vernalized variants, manifested by r-values 0.55 and 0.54 (p-values 5.0 × 10^–15^ and 9.5 × 10^–15^) for non-vernalized plants, followed by r-values 0.39 and 0.40 (p-values 6.6 × 10^− 8^ and 5.5 × 10^− 8^) for vernalized ones. A third largest cluster, C5 carrying 28 accessions, was significantly correlated with late phenotype of non-vernalized plants, however, with remarkably lower parameters (r-values 0.27 and 0.28, p-values 0.0004 and 0.0002) than the two previously mentioned clusters (Fig. 3). Moreover, an *L. hispanicus* accession HIS_232 collected in Greece formed a separate cluster (C2) together with two other accessions with unknown origin (HIS_240 and HIS_241), indicating possible evolutionary diversification by geographic isolation (Fig. 2).

### GWAS highlighted several loci significantly associated with *L. hispanicus* phenology and vernalization responsiveness

Based on the PCA and cross-entropy analysis, K = 6 was selected as the representative number of clusters for population structure in GWAS (Supplementary Figures S1 and S2). Two years (2022 and 2023) and two experimental variants, non-vernalized (N) and vernalized (V) were analyzed. Analyzed trait included the number of days from sowing to the floral bud emergence (NB_2022, NB_2023, VB_2022 and VB_2023), start of flowering (NF_2022, NF_2023, VF_2022 and VF_2023) and pod maturity (NP_2022, NP_2023, VP_2022 and VP_2023). Moreover, the number of days was recalculated as the cumulative number of growing degree days until the floral bud emergence (traits NB_GDD_2022, NB_GDD_2023, VB_GDD_2022 and VB_GDD_2023), start of flowering (NF_ GDD_2022, NF_ GDD_2023, VF_ GDD_2022 and VF_ GDD_2023) and pod maturity (NP_ GDD_2022, NP_ GDD_2023, VP_ GDD_2022 and VP_ GDD_2023). The same recalculation was done for the total photoperiod hours, providing another set of traits (NB_H_2022, NB_H_2023, VB_H_2022, VB_H_2023, NF_ H_2022, NF_ H_2023, VF_ H_2022, VF_ H_2023, NP_ H_2022, NP_ H_2023, VP_ H_2022 and VP_ H_2023, respectively). In total 36 variables were analyzed in GWAS. As 25.4% markers did not match any of the *L. luteus* scaffolds, we compared the results obtained with two algorithms, FarmCPU and BLINK, as the first one accounts the genomic position of markers whereas the second one does not analyze it.

The total number of 78 markers revealed at least one marker-trait association with FDR-corrected P-value below the 0.05 threshold, including 47 markers found by BLINK, 40 by FarmCPU and 9 markers significant by both methods (Supplementary Table S5). As many as thirty three markers revealed significant associations with three variables by one of the algorithms, however, usually it was just association with the number of days for one phenological trait and with cumulative GDDs and photoperiod hours calculated for this trait. Therefore, to focus on the more universal markers we looked for the shortlisted thirteen markers showing significant BLINK or FarmCPU associations with at least four variables (Table 1). This set included twelve PAV markers and one SNP marker. Moreover, six markers from this list were identified as significantly associated by both algorithms. Minor allele frequency (MAF) ranged from 6.3% to 9.9% (markers M065429 and M055339) to 47.3%, 47.6% and 48.2% (markers N114410, M089187 and M092420). Marker significantly associated with plant phenology without vernalization were shown not significant after vernalization and vice versa. One marker, M074938, was significantly associated with plant phenology in both years for non-vernalized plants. Employing the Best Linear Unbiased Estimates (BLUEs) method within GWAS, which integrates phenotypic data across multiple years, has confirmed the significant association of four previously selected markers (M065429, M079035, M055339, and M102006), as well as revealed an additional one: M076759. Interestingly, the M076759 marker exhibits a remarkable similarity to M074938, both in terms of position on *L. luteus* chromosome YL-12 (4791451–4791519 bp vs. 4791386–4791452 bp) and allele segregation profile within our *L. hispanicus* diversity panel. The results of BLUE-based GWAS are provided in Supplementary Table S6.

**Table 1:** Markers that revealed significant associations with at least four variables in *Lupinus hispanicus* diversity panel.

Marker	L. luteus chromosome	Position	Minor allele frequency (%)	No. of BLINK associations	No of Farm CPU associations
M104942	YL-16	23 961 978	24.4	9	7
M079035	YL-23	17 472 037	25.6	5	6
M102006	YL-22	28,557,468	33.1	5	6
M074938	YL-12	4,791,386	18.7	8	2
M055339	YL-03	35,159,449	9.9	3	6
M079300	YL-26	20,608,686	24.1	6	3
M065429	YL-25	17,598,995	6.3	0	6
M089187	-	-	47.6	6	0
N114410	YL-16	7,673,803	47.3	0	6
M076759	YL-12	4,791,451	19,1	6	-
M103926	-	-	26.2	5	0
M075705	YL-04	8,662,920	23.8	0	4
M092420	YL-19	29,238,434	48.2	4	0
M103410	-	-	26.8	4	0

The effects of significantly associated markers for non-vernalized plants and the number of days to the floral bud emergence ranged from − 8.0 to 12.0 days in 2022 and from − 15.1 to 11.8 days in 2023, whereas for the start of flowering ranged from − 16.5 to 12.0 days in 2022 and from − 12.4 to 9.5 days in 2023. Corresponding values for GDDs until the floral bud emergence reached from − 181.3 to 283.4 in 2022 and from − 247.4 to 231.4 in 2023, whereas for GDDs until flowering ranged from − 392.6 to 219.0 and from − 315.2 to 204.6, respectively. Similarly, these effects for the total photoperiod hours until the floral bud emergence ranged from − 236.0 to 154.4 h in 2022 and from − 186.8 to 174.4 h in 2023, whereas until flowering reached values from − 348.9 -in 2022 to 146.2 h in 2023.

**Fig. 4:**
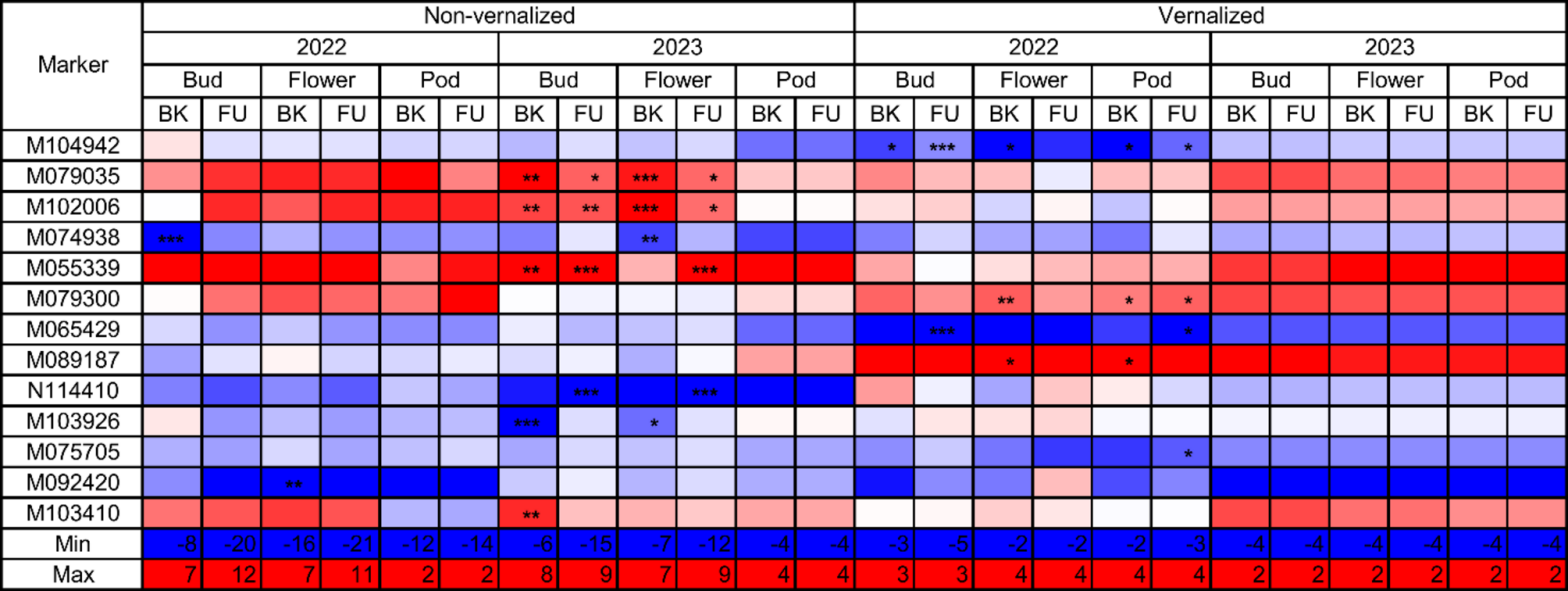
Marker effects for 13 markers significantly associated with at least four variables. Observed traits include the number of days from sowing to the floral bud emergence, start of flowering and pod maturity for non-vernalized and vernalized plants. Phenotypic observations were performed during 2022 and 2023 growing seasons in a greenhouse at the Institute of Plant Genetics, Polish Academy of Sciences, Poznań, Poland (52°26′ N 16°54′ E). Color scale is presented from blue (minimum value) through white (zero) to red (maximum value). BK stands for BLINK, whereas FU for FarmCPU.

For vernalized plants, significantly associated markers were identified only for the year 2022, but also for the pod maturity. The effects of those markers ranged from − 4.6 to 2.0 for the number of days from sowing until the floral bud emergence, from − 2.9 to 4.3 days for flowering, and from − 3.3 to 4.1 days for the pod maturity. Effects for GDDs reached from − 81.3 to 47.9 for the floral bud emergence, from − 48.1 to 87.5 for flowering and from − 58.2 to 78.9 for the pod maturity, whereas effects for the total photoperiod hours ranged from − 72.3 to 30.7, from − 46.7 to 68.6 and from − 57.2 to 67.8, respectively. Visualization of effects for the number of days from sowing to the floral bud emergence, start of flowering and pod maturity was provided on Fig. 4. Circular visualizations of Manhattan plots for GWAS were provided in Supplementary Figure S3.

**Fig. 5:**
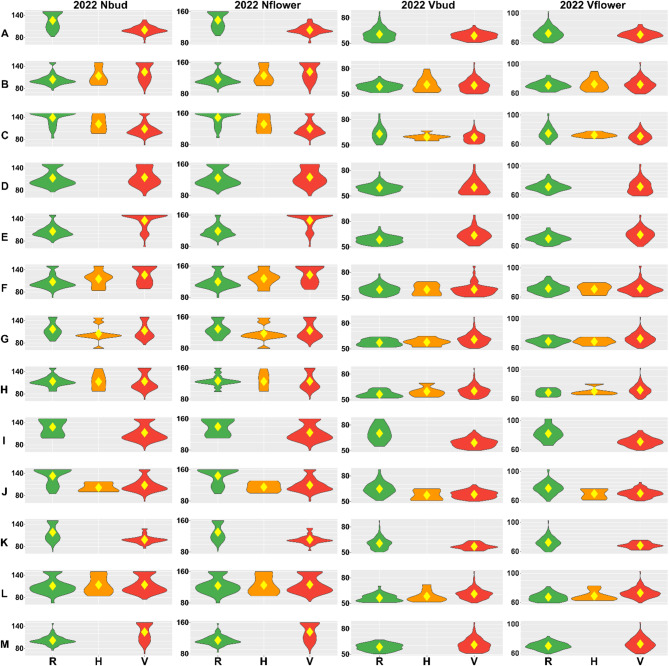
Allelic effects on the number of growing degree days (GDDs) for thirteen DArT-seq markers, M092420 (**A**), N114410 (**B**), M074938 (**C**), M103926 (**D**), M103410 (**E**), M102006 (**F**), M079035 (**G**), M055339 (**H**), M065429 (**I**), M075705 (**J**), M104942 (**K**), M079300 (**L**), and M089187 (**M**) tagging novel QTL regions. R stands for the reference allele (**0**), V for an alternative allele (2), whereas H for a heterozygote (1). Observed traits include the number of days from sowing to the floral bud emergence (bud), start of flowering (flower) and pod maturity (pod) for non-vernalized (Nbud, Nflower, Npod) and vernalized plants (Vbud, Vflower, Vpod). Phenotypic observations were performed during 2022 growing season in a greenhouse at the Institute of Plant Genetics, Polish Academy of Sciences, Poznań, Poland. Diamonds indicate mean values.

**Fig. 6:**
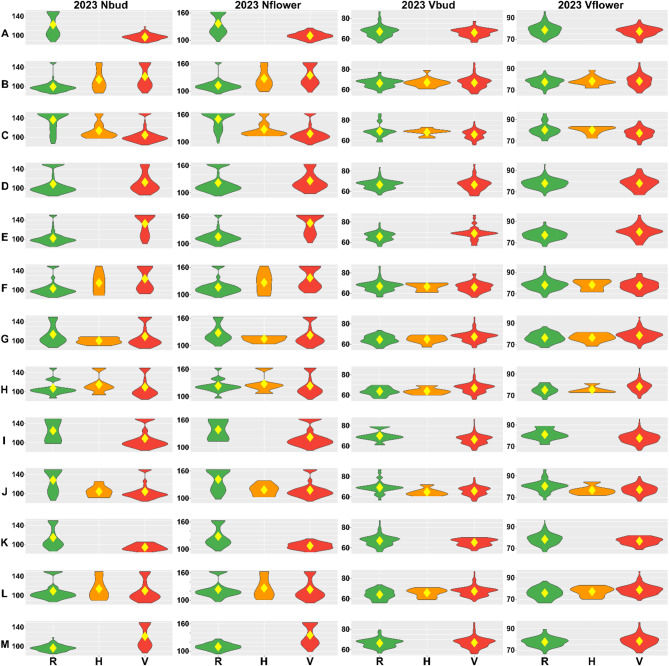
Allelic effects on the number of growing degree days (GDDs) for thirteen DArT-seq markers, M092420 (**A**), N114410 (**B**), M074938 (**C**), M103926 (**D**), M103410 (**E**), M102006 (**F**), M079035 (**G**), M055339 (**H**), M065429 (**I**), M075705 (**J**), M104942 (**K**), M079300 (**L**), and M089187 (**M**) tagging novel QTL regions. R stands for the reference allele (**0**), V for an alternative allele (2), whereas H for a heterozygote (1). Observed traits include the number of days from sowing to the floral bud emergence (bud), start of flowering (flower) and pod maturity (pod) for non-vernalized (Nbud, Nflower, Npod) and vernalized plants (Vbud, Vflower, Vpod). Phenotypic observations were performed during 2023 growing season in a greenhouse at the Institute of Plant Genetics, Polish Academy of Sciences, Poznań, Poland. Diamonds indicate mean values.

Besides the effects reported by GWAS accounting for population structure, we analyzed the distribution of GDDs recorded for both alleles and heterozygotes of selected 13 significant markers in the germplasm diversity panel (Figs. 5 and 6). The direction of GDD effects for an alternative allele was coherent between years and traits for all markers in non-vernalized plants and for all markers except M074938 and M089187 in vernalized plants. Heterozygotes revealed phenotypes intermediating between those observed for the two opposite homozygotes, indicating incomplete dominance. It should be noted direction of allelic effects reported by BLINK and FarmCPU programs (Fig. 4) were coherent with GDD effects in germplasm diversity panel for all significant marker-trait associations (Figs. 5 and 6). However, for some non-significant marker-trait associations in non-vernalized plants, they were opposite (i.e. markers N114410, M103926, M079035 and M055339).

**Fig. 7:**
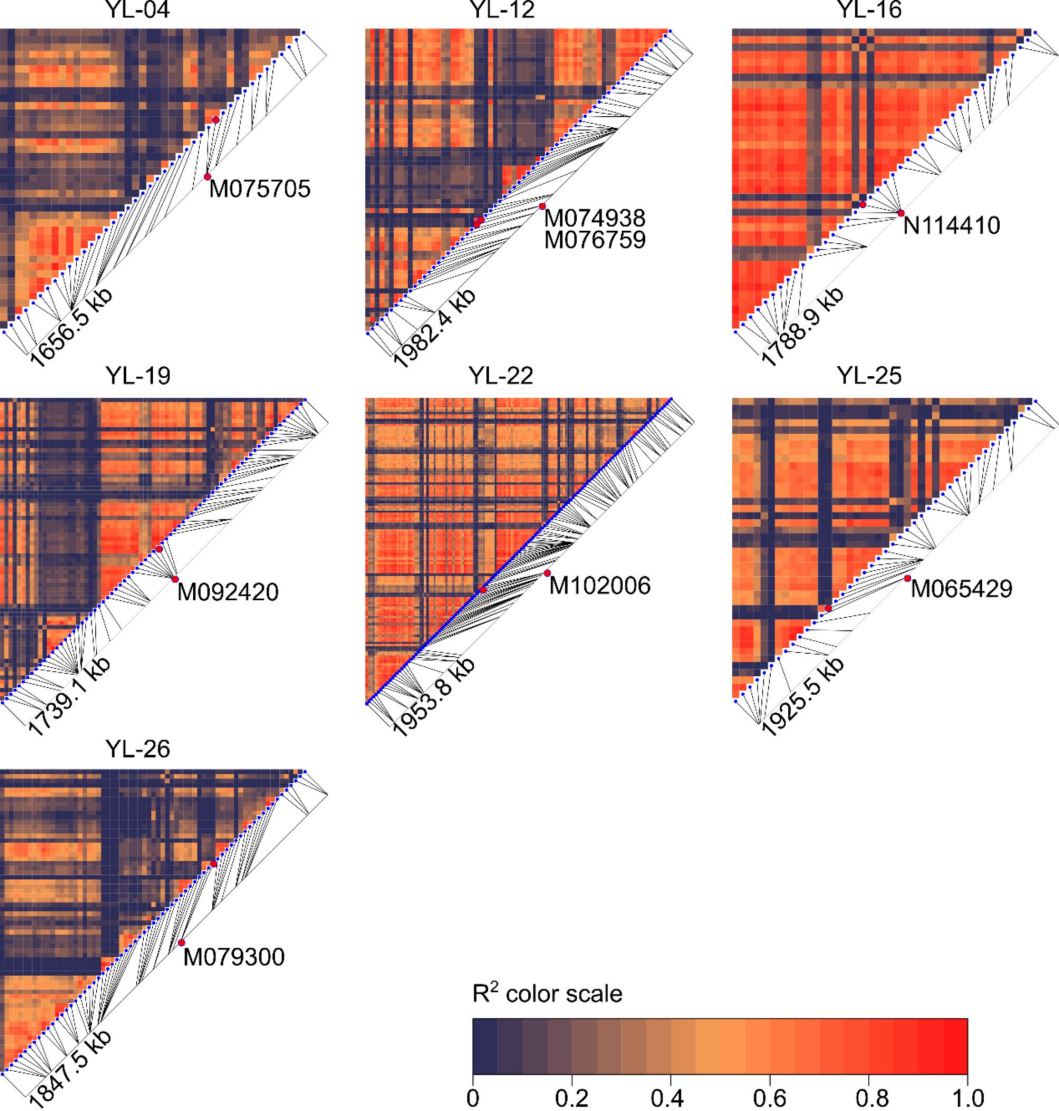
Linkage disequilibrium (LD) plots for *L. luteus* genomic regions carrying DArT-seq markers significantly associated with *L. hispanicus* plant phenology. The pairwise r2 values between markers are shown. Red indicates high measures of LD, while deep violet indicates low LD. Lengths of analyzed segments is provided on the marker bars.

As the majority of *L. hispanicus* DArT-seq markers were localized on the pseudochromosomes of the closely related species *L. luteus*, we were able to analyze linkage disequilibrium (LD) decay around marker loci. From the 14 markers significantly associated with at least four variables in GWAS (including an additional one from BLUE analysis), eight were located in regions well saturated with other DArT-seq loci (≥ 20 markers per 1 Mbp) and as such were subjected to LD analysis (Fig. 7).

Markers N114410 from chromosome YL-16, M092420 from YL-19 and M102006 from YL-22 were localized in the middle of the regions exhibiting high LD values on large (> 1 Mbp) chromosomal segments. Markers M074938 from chromosome YL-12, M065429 from YL-25 and M079300 from YL-26 showed rapid LD decay with chromosomal distance, whereas a marker M075705 from chromosome YL-04 revealed intermediate values of LD. However, rapid LD decay around some marker loci may result from using a proxy *L. luteus* genome for sequence alignments and the possibility of read mapping into incorrect locations or the presence of recombination hotspots causing break of microsynteny between these two species.

### Candidate genes identified by shared microsynteny

Due to the lack of sequenced *L. hispanicus* genome and raw annotation of pre-publication *L. luteus* genome release (GCA_964019355.1), we exploited shared microsynteny between *L. luteus* scaffolds carrying significant *L. hispanicus* markers and the reference *L. albus* pangenome assembly to identify candidate genes for markers showing significant BLINK or FarmCPU associations with at least four variables^50,51^. Alignment of DArT-seq sequences revealed gene-specific hits for all 14 selected markers in the *L. albus* genome and for all markers except M104942 in the *L. luteus* genome. According to the annotations provided in the *L. albus* genome assembly, those genes putatively encode such proteins as Zf-FLZ domain-containing protein, pre-mRNA polyadenylation factor Fip1, pectinesterase/pectinesterase inhibitor 41, ribosomal protein L34Ae, cellulose synthase (UDP-forming), transcription initiation factor IIB, Pumilio RNA binding domain-containing protein, cytochrome P450, DNA helicase, serine/threonine kinase, ALP1-like protein, and two uncharacterized proteins (Table 2). Besides genes directly tagged by DArT-seq marker alignments, some neighboring sequences that could be present in the same LD blocks were also chosen. Those included putative transcription factors from bZIP, MYB-HB-like, C2H2, MADS-MIKC and WD40-like families, Zf-FLZ domain-containing protein, replication termination factor 2 RTF2, small and long non-coding RNAs, mir-393 precursor and other putative regulatory sequences. Their potential involvement in flowering regulatory pathways and putative association with flowering induction is presented in Discussion. Finally, we conducted an analysis of microsynteny shared between *L. luteus* and *L. albus* genome assemblies in regions adjacent to the selected significantly associated DArT markers. The lack of microsynteny between *L. albus* and *L. luteus* was observed only for the genome region carrying M092420 marker sequence, where two neighboring genes located *L. albus* chromosome 25, *Lalb_Chr25g0286361* and *Lalb_Chr25g0286351*, were mapped to loci localized on two different chromosomes in *L. luteus*, namely LLUT_LOCUS27614 on chromosome YL-19 and LLUT_LOCUS11214 on chromosome YL-07. Moreover, an *L. luteus* LLUT_LOCUS18135 gene on chromosome YL-12 carrying M089187 marker sequence aligned to two genes in *L. albus* genome, Lalb_Chr06g0173461 and Lalb_Chr21g0316521. The latter one revealed higher sequence similarity to LLUT_LOCUS2261 on different chromosome (YL-15) to the original LLUT_LOCUS18135 (Table 2).

**Table 2:** Genetic annotation of selected DArT-seq markers, highlighted by GWAS as significantly associated. Two lupin genomes (*L. luteus* and *L. albus*) were used for BLAST mapping of DArT-seq markers. Loci directly matching DArT-seq markers are indicated by asterisks, whereas corresponding neighboring loci (if applicable) are listed in row(s) below them.

Marker	L. luteus gene	L. albus gene	Annotation
M104942	N/D	Lalb_Chr14g0365811*	non-coding RNA
M104942	N/D	Cluster_73519	non-coding RNA
M104942	N/D	Lalb_Chr14g0365831	Putative transcription factor bZIP family/TGA2.3-like
M079035	LLUT_LOCUS32250*	Lalb_Chr09g0329621*	Putative Zf-FLZ domain-containing protein
M079035	N/D	Cluster_49371	long non-coding RNA
M102006	LLUT_LOCUS31495*	Lalb_Chr13g0300951*	Putative pre-mRNA polyadenylation factor Fip1
M102006	LLUT_LOCUS31494	Lalb_Chr13g0300941	Putative Ubiquitin domain, replication termination factor 2 RTF2, Zinc finger, RING/FYVE/PHD-type
M074938	LLUT_LOCUS17529*	Lalb_Chr02g0146461*	Putative pectinesterase/pectinesterase inhibitor 41
M055339	LLUT_LOCUS4667*	Lalb_Chr03g0032501*	Unknown/hypothetical protein
M055339	LLUT_LOCUS4666	Lalb_Chr03g0032521	Putative transcription factor bZIP/ABA INSENSITIVE 5-like protein 2
M055339	LLUT_LOCUS4656	Lalb_Chr03g0032571	Putative transcription factor MYB-HB-like family/GAMYB-like
M079300	LLUT_LOCUS36319*	Lalb_Chr01g0012161*	Putative ribosomal protein L34Ae
M079300	LLUT_LOCUS36317	Lalb_Chr01g0012131	Putative transcription factor C2H2 family/zinc finger protein 1-like
M065429	LLUT_LOCUS34632*	Lalb_Chr19g0132221*	Putative cellulose synthase (UDP-forming) chromatin regulator PHD family
M065429	LLUT_LOCUS34631	Lalb_Chr19g0132201	Putative transcription factor C2H2 family/E3 ubiquitin ligase BIG BROTHER
M089187	LLUT_LOCUS18135*	Lalb_Chr06g0173461*	Transcription initiation factor IIB
M089187	LLUT_LOCUS22611	Lalb_Chr21g0316521	Putative transcription factor MADS-MIKC family/ Agamous-like MADS-box protein AGL18
N114410	LLUT_LOCUS23544*	Lalb_Chr14g0368451*	Putative armadillo-like helical, pumilio, RNA binding domain-containing protein
N114410	LLUT_LOCUS23547	Lalb_Chr14g0368401	Putative transcription factor MYB-HB-like family/MYB44-like
M076759	LLUT_LOCUS17529*	Lalb_Chr02g0146461*	Putative pectinesterase/pectinesterase inhibitor 41
M103926	LLUT_LOCUS4442*	Lalb_Chr03g0034641*	Putative cytochrome P450/MFP1 attachment factor
M103926	N/D	Lalb_Chr03g0034631	mir-393 microRNA precursor family
M075705	LLUT_LOCUS5864*	Lalb_Chr11g0074201*	Putative DNA helicase/ATP-dependent DNA helicase Q-like 3
M075705	LLUT_LOCUS5876	Lalb_Chr11g0074111	Putative Zf-FLZ domain-containing protein/FCS-Like Zinc finger 13
M092420	LLUT_LOCUS27614*	Lalb_Chr25g0286361*	Putative protein-serine/threonine kinase CMGC-CDK-CRK7-CDK9 family
M092420	LLUT_LOCUS11214	Lalb_Chr25g0286351	Putative protein kinase PEK-PEK family transcription factor WD40-like family/SPA1-RELATED 4-like
M103410	LLUT_LOCUS13188*	Lalb_Chr05g0219221*	Antagonist of like heterochromatin protein 1-like

## Discussion

### DArT sequencing is an effective method for genotyping non-model plants

Diversity Array Technology, DArT, is a sequence-independent, microarray-based, a high-throughput genome analysis method that enables multiplexing and allows the simultaneous interrogation of several hundred to several thousand polymorphic loci across the genome. This type of polymorphism is based on sequence variations and methylation patterns at the recognition sites of restriction enzymes (REs) and insertions or deletions (INDELS). REs-based methods provide greater accuracy, resulting in improved reproducibility compared to PCR-based methods, which rely on the lower precision from selective primer binding^52,53^. It was demonstrated that the vast majority of sequenced DArT markers (about 97%) scored identical, irrespective of chosen DNA preparations, but the remaining 3% gave consistently different results for different DNA samples. It suggests that a marginal effect of DNA methylation on the outcome needs to be considered^54^. Studies on white lupin and narrow-leafed lupin have demonstrated that, although rearrangements in the promoter regions of *FT* genes have a significant impact, none of the DArT markers have been found near their sequences^23,41,55,56^. This is likely due to the conserved nature of promoter regions, which results in a lack of suitable cleavage sites for restriction enzymes and selective pressure against polymorphisms in these areas. As a result, the likelihood of detecting DArT markers in promoter regions is lower compared to other parts of the genome, such as introns or intergenic regions. Nonetheless, the DArT markers have been integrated into analyses of many plant species, including major crops like wheat, barley, common bean, and rice, as well as non-model plants such as lupin^23,54,57,58,59,60^. They have been employed extensively for the construction of molecular maps, identifying trait-marker associations, assessment of genetic diversity, association mapping, and routine genotyping in various crops for varietal identification^60,61,62,63,64,65^.

### Perspectives for adaptation of *L. hispanicus* for spring and autumn sowing in Europe

Greenhouse observations performed in the study revealed high variability of flowering time and vernalization responsiveness in *L. hispanicus*, highlighted by wide range of days to flowering of non-vernalized plants (from 79.3 to more than 160 days) and significant differences between accessions in acceleration of flowering by vernalization treatment (from 15.2 to more than 93 days). The number of days from sowing to flowering of non-vernalized late *L. hispanicus* accessions observed in the present study converges with previously reported values of 143, 164–183, and 211 days^12,14,66^. Early and low-responsive to vernalization accessions of *L. hispanicus* were not reported previously to our knowledge. Georeferenced records localize *L. hispanicus* specimens in Iberian Peninsula regions with climatic conditions enabling effective vernalization during winter (https://www.gbif.org/species/2964255), including regular occurrence of mild frost (a few °C below zero) in many locations^14,67,68^. A large number of *L. hispanicus* locations experience more than 1200 chilling hours during typical winter^69^. A value of 1200 chilling hours corresponds to about 50 days of effective vernalization, well above the three-week period required for Old World lupins to be physiologically vernalized^22,23,24,35,55^. Due to the very frequent occurrence of late spring and early summer droughts in the Iberian Peninsula climate, *L. hispanicus*, like many other plant species, evolved a drought escape strategy by autumn seed germination, winter growth, and significant spring phonological response to winter vernalization towards flowering and yield production^70^. Such a strong response to vernalization has been demonstrated in our study by a much shorter range and lower values of growing degree days (GDDs) from sowing to the start of flowering with applied vernalization treatment (from 1018 to 1882 GDDs) than without it (from 1412 to 3211 GDDs). Similar experiments performed for *L. albus* revealed the values from sowing to start of flowering in the range from 698 to 2397 GDDs without vernalization and from 518 to 1435 GDDs with vernalization^23^. These values are lower than those measured for *L. hispanicus*, however, vernalization responsiveness in some late flowering *L. albus* accessions was also as sound as in *L. hispanicus*. Based on the reported number of days from sowing to flowering, we were able to calculate GDDs also for two other Old World lupin crop species, *L. angustifolius* and *L. luteus*^22,55^. This calculation revealed that *L. angustifolius* flowered in Poznań 716 to 2250 GDDs after sowing without vernalization and 640 to 1164 GDDs after vernalization treatment, whereas for *L. luteus* these values were 873–1816 GDDs and 763–1709 GDDs, respectively (Table 3). Comparison of those values confirmed reported much later phenology of *L. hispanicus* than all domesticated Old World lupin species and revealed its significantly higher vernalization responsiveness^12,14,66^. Nevertheless, the earliest *L. hispanicus* lines could be placed in the middle of the GDDs range reported for lupin cultivars, intermediating between winter- and spring ecotype.

To verify if *L. hispanicus* GDDs values correspond to the climatic variables conditioning lupin spring sowing in western Poland, we calculated GDDs for 2022 and 2023 growing seasons in Poznań using meteorological data reported by the Institute of Meteorology and Water Management - National Research Institute in Poland (https://danepubliczne.imgw.pl/pl). Assuming spring sowing on the 1st April, the threshold of 2000 GDDs was reached on 24th August in 2022 and 18th August in 2023, whereas with sowing on 1st May (to avoid common spring frosts), this threshold was achieved on the beginning of September in both years^71,72^. Therefore, expected performance of non-vernalized early *L. hispanicus* accessions in this location could converge with the moderately late accessions of soybean, yielding in middle autumn, when weather may become less favorable for pod drying^73^. It should be noted that there is a significant SW-NE decreasing GDDs gradient in Poland, following transition from oceanic to humid continental climate^74^. Taking into consideration the whole European continent, a clear latitudinal pattern of GDDs has been observed, with the highest values, recalculated to the base temperature of 3 °C, exceeding 4000 GDDs in the Mediterranean and the Black Sea regions^75^. Therefore, the earliest *L. hispanicus* accessions should develop seed yield across a wide range of European environments, even if sown in April/May and cultivated in temperatures above vernalization threshold for the whole growing period, if properly watered.

On the contrary, intermediate and, particularly, late *L. hispanicus* accessions will have to be subjected to at least partial vernalization, even in the warmest regions of the continent, to ensure timely flowering and dry pod harvesting. It can be achieved by autumn sowing, however, artificial adaptation of *L. hispanicus* to harsh winters outside mild Mediterranean climate zone requires selection towards frost resistance. It should be some frost resistance that had already evolved in natural *L. hispanicus* populations, because plant annual communities growing on the semi-arid Mediterranean steppe in Spain exhibited very good performance until frost reaching − 4.0 °C, whereas the lethal temperature causing 50% frost damage (LT50) was relatively low (about − 9.5 °C)^68^. Recent evaluation of *L. albus* frost tolerance using high-throughput phenotyping platform revealed that the most winter-adapted accessions have the 4-hours LT50 value about − 12.0 °C, however, with significant visual biomass injury starting from − 9 °C^76^. Preceding study encompassing all three Old World lupin crop species reported moderate frost tolerance of winter *L. albus* and *L. angustifolius* cultivars (up to -10 °C, so well-aligned with both studies) and low frost tolerance of *L. luteus*, albeit without information about *L. luteus* ecotypes used in the study (spring or winter)^22,34,77^. As vernalization responsiveness is highly correlated with frost tolerance in *L. albus*, it can be expected that late flowering *L. hispanicus* accessions that are highly responsive to vernalization will have higher frost tolerance than the early ones. Reaching the frost visual damage threshold value similar to that reported for winter ecotypes of *L. albus* probably will enable *L. hispanicus* yielding as a winter crop in the large part of the European continent^76^.

**Table 3:** Comparison of the total number of growing degree days (GDDs) from sowing to flowering between *L. hispanicus* and three old world domesticated lupin species (*L. luteus*, *L. albus* and *L. angustifolius*) in greenhouse (CE) and field (F) conditions^22,55^.

Species	Year	Vernalization	Environment	Mean GDDs	Min GDDs	Max GDDs
*L. hispanicus*	2022	—	CE (Poznań)	2410	1412	3177
	2022	+ +	CE (Poznań)	1262	1018	1882
	2023	—	CE (Poznań)	2362	1700	3211
	2023	+ +	CE (Poznań)	1376	1151	1744
*L. luteus*	2016	—	CE (Poznań)	1283	927	1816
	2016	+ +	CE (Poznań)	1183	918	1566
	2017	—	CE (Poznań)	1220	873	1745
	2017	+ +	CE (Poznań)	1078	777	1510
	2019	—	CE (Poznań)	1268	889	1742
	2019	+ +	CE (Poznań)	1116	763	1709
*L. albus*	2020	—	CE (Poznań)	1308	726	2397
	2021	—	CE (Poznań)	1229	698	2283
	2004	+ +	F (Lodi)	870	651	1048
	2005	+	F (S. Sauvant)	698	518	1100
	2004	+	F (Sanluri)	996	816	1435
*L. angustifolius*	2014	—	CE (Poznań)	1340	716	2250
	2014	+ +	CE (Poznań)	813	640	1164

The present study revealed significant association of *L. hispanicus* phenology with population structure, manifested by high correlations between assignment to particular clusters and the number of days from sowing to floral bud emergence, start of flowering and pod maturity. Similar observation was made for *L. albus* where early flowering genotypes from East African resources (Ethiopia, Kenya and Sudan), Egypt, Maghreb (Morocco) and West Asia (Israel and Syria) grouped together, whereas late flowering Azorean, Madeiran and Canarian genotypes also formed a separate cluster^23^. Moreover, such diversification was also found in two other Old World domesticated species, *L. angustifolius* and *L. luteus*, where Palestinian accessions revealed distinct phenology^24,78^.

### Candidate genes controlling *L. hispanicus* phenology

Alignment of markers that revealed significant associations in BLINK or FarmCPU with at least four variables to the genomes of closely related species, *L. luteus* and *L. albus*, enabled us to search for candidates genes by analysis of microsyntenic blocks shared between all three species. The M102006 marker, especially sound in non-vernalized plants, aligned to the LLUT_LOCUS31495 gene on YL-22 and putative pre-mRNA polyadenylation factor Fip1 (Lalb_Chr13g0300951) gene on WL-13. The FIP1, Factor Interacting with Poly(A) Polymerase 1, a component of the pre-mRNA 3’ end processing machinery and in *Arabidopsis* plays a significant role in the processes of seed dormancy and germination. FIP1 is predominantly expressed in seeds, and the knockout of FIP1 results in decreased seed dormancy and insensitiveness to exogenous abscisic acid (ABA) during seed germination and the early stages of seedling establishment^79^. Furthermore, the region of the WL-13 chromosome located ~ 100 kb from the current marker, was highlighted in a previous study to carry the Chr13_13913452_D marker significantly associated with flowering induction in *L. albus*^23^. Moreover, the major QTL for flowering time identified in this species was also located here^60,80,81,82^. One PAV marker from this region, Chr13_12561729_D, was successfully transformed to a PCR marker and validated in a large *L. albus* germplasm pool to be significantly associated with flowering time in the absence of vernalization^64^.

An M079035 marker, that showed similar effects as M102006 marker (Fig. 4), was mapped to the LLUT_LOC32250 gene on YL-23 in *L. luteus* and to a gene encoding a putative Zf-FLZ domain-containing protein (Lalb_Chr09g0329621) on WL-09 in *L. albus*. BLAST analysis of both genes revealed considerable similarity to the transcription factor FCS-Like, which in *A. thaliana* exhibits high expression levels in reproductive organs, such as flowers. This observation may indicate its potential role in the regulation of organ development or transition between vegetative and reproductive phases^83^.

The next marker, M074938 was the only marker significantly associated with non-vernalized plants in both 2022 and 2023. Moreover, the BLUE-based GWAS which combined 2022 and 2023 phenology revealed only one new marker, M076759 which demonstrated a remarkable similarity to M074938, both in terms of position on *L. luteus* chromosome and allele segregation profile. Both these markers were mapped to loci on two, different chromosomes in *L. luteus* (YL-12 and YL-01) and *L. albus* (WL-06 and WL-02). All highlighted sequences demonstrated the highest similarity to the pectinesterase inhibitor 41 (PMEI41), which may play a role in post-transcriptional regulation. In transgenic *Arabidopsis* plants, under both long-day and short-day photoperiods, PMEI2 and PMEI4 facilitated early flowering phenotypes, accompanied by elevated expression levels of multiple flowering-related genes^84^. Notably, in WL-02, this marker was located approximately 600 kb from two significant markers, Chr02_2625514_D and Chr02_2625564_D, which delimit the second major flowering time QTL in *L. albus*^60^^80^^82^.

Another marker associated with non-vernalized plants flowering, M055339, has been identified in the YL-03 and WL-03 regions, with both instances classified as uncharacterized transcripts or hypothetical proteins. Notably, the adjacent genes in *L. luteus*, located 4 kb downstream (LLUT_LOCUS4666), and in *L. albus*, positioned 6 kb upstream (Lalb_Chr03g0032521), were annotated as ‘Putative transcription factor bZIP family.’ These genes exhibit the highest similarity to ABA INSENSITIVE 5-like protein 2, which plays a significant role in regulating plant growth and development^85^.

An M103926 marker significant for non-vernalized plants was mapped with relatively low sequence identity of ~ 84% in YL-12 and WL-03. Both loci demonstrate similarity to Cytokinin hydroxylase-like/Putative cytochrome P450 gene. Notably, this hit is situated within a microRNA-rich region of *L. albus*, with the nearest microRNA cluster identified as the mir-393 microRNA precursor. In *Arabidopsis*, miR393a and miR393b target F-box auxin receptors TIR1 (TRANSPORT INHIBITOR RESPONSE 1), AFB1 (AUXIN SIGNALING F BOX PROTEIN 1), AFB2, and AFB3 ^86^. The overexpression of miR393 led to a decrease in TIR1 mRNA levels; conversely, the miR393-resistant variant of TIR1 (mTIR1) caused an accumulation of mTIR1 transcripts, which reportedly induced delayed flowering in *Arabidopsis*, potentially by modifying the expression of various auxin-responsive genes^87^.

The most significant BLAST results for marker M092420, which has been mapped to YL-19 and WL-25, were identified as members of the putative protein-serine/threonine kinase family. Notably, the closest gene, located approximately 1 kb downstream in *L. albus*, has been annotated as a putative protein kinase from the PEK-PEK family and resembles a WD40-like transcription factor. This gene exhibits the highest similarity to SPA-1-RELATED 4-like proteins in *Arabidopsis*, which are known to regulate photoperiodic flowering by interacting with *CO* to modulate its stability^88^. Furthermore, this significant hit is situated within a previously characterized region of WL-25 associated with flowering time^23^.

The last marker for flowering of non-vernalized plants that matched flowering-related genes by microsynteny, M103410, mapped to YL-09 and WL-05. A BLAST analysis of both locations revealed the highest similarity with ANTAGONIST OF LIKE HETEROCHROMATIN PROTEIN 1, ALP1. In *Arabidopsis*, ALP2, in conjunction with ALP1, demonstrates a reduced expression of floral identity genes such as PI, AG, and SEP3, indicating a role for ALPs in floral induction. Furthermore, protein interaction assays indicate that ALP2 binds directly with MSI1 and is essential for the interaction between ALP1 and PRC2^89^.

The M104942 marker, associated with flowering time of vernalized plants, aligned to the non-coding region of *L. luteus* chromosome YL-16 and the *L. albus* chromosome WL-15 region carrying the long non-coding RNA cluster (Lalb_Chr14g0365811). The nearest neighboring gene, positioned ~ 2.5 kb upstream, is a putative transcription factor from the bZIP family (Lalb_Chr14g0365831), identified by BLAST analysis as the TGA2.3-like transcription factor. It has been already demonstrated that TGA transcription factors can influence flowering time, such as *Arabidopsis thaliana AtTGA4* that directly interacts with the promoter region of *CONSTANS* (*CO*), a key regulator of the *Flowering locus T* (*FT*). Furthermore, the functional loss of AtTGA7 delayed Arabidopsis flowering by altered expression of the critical flowering inhibitor gene, *FLC*^90^.

The following marker associated with flowering of vernalized plants, M079300, localized to LLUT_LOCUS36319 gene on YL-26 that did not match any *L. albus* gene with known involvement in regulation of flowering. However, the adjacent LLUT_LOCUS36318 gene, located 7 kb downstream, mapped in *L. albus* ~ 6 kb from a putative transcription factor from the C2H2 family, which is highly similar to Zinc Finger Protein 1-like (ZFP1-like). This protein, along with ZFP8, is known to be down-regulated by LEAFY and APETALA1, thereby alleviating their repression of class B and C floral homeotic genes^91^.

An M089187 marker, also associated with vernalized plant flowering, yielded one signal in *L. luteus* chromosome YL-12 and two hits in *L. albus*, localized on the WL-06 and WL-21 chromosomes. The hits corresponding to YL-12 and WL-06 exhibited a high degree of similarity with the Transcription Initiation Factor IIB. In contrast, the hit on WL-21 was located 10 kb from a Putative Transcription Factor of the MADS-MIKC family (Lalb_Chr21g0316521), highly similar to the AGAMOUS-like MADS-box protein AGL18. AGL18 functions as a transcription factor that plays a crucial role in the negative regulation of flowering via the photoperiodic pathway^92^.

Another marker that was significantly correlated for vernalized plants, M075705, mapped on YL-04 and WL-11 chromosomes, aligned to DNA helicases. Approximately 60 kilobases downstream from this marker on the WL-11 chromosome, there are two copies of putative Zf-FLZ domain-containing proteins (Lalb_Chr11g0074111 and Lalb_Chr11g0074121), identified as *FLZ13*. These proteins are known to interact with *FLC* and *ABI5*, thereby negatively regulating flowering time in *Arabidopsisf*^93^.

We were unable to identify good candidate genes for the markers M065429 and N114410, localized in *L. luteus* genome on chromosomes YL-25 and YL-16. It is likely that M065429 marker is situated in region that is not conserved between *L. hispanicus* and *L. luteus* genomes, because we found rapid LD decay around this locus (Fig. 7). It could explain the lack of hits to known genes involved in flowering regulation as there is putatively no microsynteny here. However, this explanation cannot be applied for the second marker, N114410, located in the middle of the large region with high LD values. The influence of factors beyond gene-mediated regulation, such as miRNA and long non-coding RNA (lncRNA) cannot be ruled out here.

## Conclusions

The phenology of the earliest *L. hispanicus* accessions matches the middle of the range of growing degree days reported for existing Old World lupin cultivars, bridging the gap between winter and spring ecotypes. These early accessions are expected to perform similarly to moderately late soybean accessions, yielding in early to mid-autumn, exploiting the full length of the growing season, and progressively expanding in the era of changing climate. Given these factors, Spanish lupin may be considered a strong candidate for the development of new cultivars, providing a valuable addition to already cultivated species. It has the potential to produce significantly more pods on the main stem, outyielding current lupin crops. However, essential agronomic traits, such as disease resistance and alkaloid content, must be improved during the domestication process.

This study identified several molecular markers significantly associated with multiple observed traits. This finding was obtained through BLINK and FarmCPU GWAS and supported by LD analysis. These markers are located in microsyntenic blocks shared between yellow and white lupin species and carry genes associated with flowering induction, flower formation, vernalization response, and photoperiod control. Due to their association with a wide range of flowering time, the identified molecular markers can be utilized in future breeding efforts to select genetic material for developing new varieties adapted to local agroclimatic conditions.

## Materials and methods

### *L. hispanicus* germplasm diversity panel

The plant material encompassed 173 accessions obtained under the Standard Material Transfer Agreement (SMTA) from five gene banks: (1) El Centro de Investigaciones Científicas y Tecnológicas de Extremadura (CICYTEX), Guadajira, province of Badajoz, Spain; (2) Genebank Gatersleben of the Leibniz Institute of Plant Genetics and Crop Plant Research (IPK), Gaterslaben, Germany; (3) United States Department of Agriculture, Agricultural Research Service, Western Regional Plant Introduction Station, Washington State University, Pullman, Washington, United States; (4) Australian Grains Genebank, The State of Victoria Department of Environment, Energy and Climate Action, Victoria, Australia; and (5) Poznan Plant Breeders Ltd., Wiatrowo Plant Breeding Station, Wiatrowo, Poland. The investigated germplasm diversity panel consisted of 107 wild representatives, 32 landraces, 3 cultivars, and 31 accessions with unknown status in the passport data. The list of accessions is provided in Supplementary Table S7.

### Seeds vernalization

The vernalization was performed on all investigated accessions using the procedure developed for other lupin species^81^. In each phenotyping experiment, half of the seeds used were vernalized on Petri dishes with moist paper at 5 °C for 21 days in 2022 and 14 days in 2023. Vernalization was conducted in the darkness to prevent green mass and root development. Non-vernalized control plants were sown four days before the end of the vernalization procedure and grown at a temperature of ~ 22 °C (~ 10 °C above the vernalization threshold) to maintain a similar thermal time^31^.

### Phenotyping of phonological phases

Greenhouse observations were performed during the 2022 and 2023 growing seasons. Phenotyping was performed at the Institute of Plant Genetics, Polish Academy of Sciences, Poznań, Poland (52°26′ N 16°54′ E). Sowing dates for the non-vernalized plants were 13th March in 2022 and 10th March in 2023, while the vernalized plants were sown four days later (17th March in 2022 and 14th March in 2023). Automatic heating kept the minimum air temperature above 18 °C, whereas cooling was maintained by a temperature-dependent window-opening system (activated at 22 °C). The number of days from sowing to (a) the appearance of the first floral bud, (b) the appearance of the first fully colored petal (flowering), and (c) the date of harvesting mature pods from the main stem were recorded. Observations were made in three biological replicates every two days, from sowing to the harvesting of the last mature pods. The experimental design is provided in Supplementary Table S1.

### **The cumulative growing degree days (GDDs)** were calculated using the formula

$$\:GDDs={\sum\:}_{t=1}^{n}\text{m}\text{a}\text{x}(\frac{Tmax+Tmin}{2}-Tb;0)$$

Where *t* and *n* are days from sowing and the total number of days from sowing to the observed phenotypic trait (floral bud emergence, start of flowering, and pod maturity), *Tmax* and *Tmin* are daily maximum and minimum temperatures, whereas *Tb* represents the base temperature parameterized in this study for lupin as 3 °C^34,94^. GDD values for fractional days were calculated on a linear scale.

### Calculation of heritability

A statistical framework was employed using R and its advanced libraries. To account for spatial variability inherent in greenhouse data, linear mixed models (LMMs) were utilized. These models were implemented with the SpATS package, incorporating row and column effects as random factors and a spatial trend component to capture environmental heterogeneity within the greenhouse. Row and columns represent a two-dimensional arrangement of pots in the greenhouse. The spatial trend was estimated using the SAP function from SpATS, ensuring the model accurately reflected spatial variability. Heritability was estimated using a generalized broad-sense heritability formula which provides a robust measure of the proportion of phenotypic variance attributable to genetic factors

$$\:{H}^{2}=\frac{\sum\:_{i=s+1}^{m}{k}_{i}}{m-s},$$

where *ki* represents the eigenvalues derived from the genetic effects model, *m* denotes the total number of eigenvalues, and *s* indicates the number of eigenvalues that are zero due to model constraints^95^. This approach accounts for both additive and non-additive genetic variances, offering a comprehensive insight into the genetic contributions to flowering traits. Analyses were conducted on datasets stratified by year to address potential temporal variability, ensuring results were reflective of annual differences.

### DNA isolation

DNA was isolated from three biological replicates per every genotype. Each time, two young upper leaves (about 100 mg of tissue) were placed in 2 ml collection tubes (Eppendorf, Hamburg, Germany) and immediately frozen in liquid nitrogen. Homogenization of frozen plant tissue was performed using two stainless steel beads (ø 5 mm, Qiagen) placed inside the collection tubes and TissueLyser II homogenizer (Qiagen, Hilden, Germany) for 45 s at 30 rpm. DNA isolation was performed in automated station Maxwell^®^ RSC 48 Instrument (Promega, Mannheim, Germany) using Maxwell^®^ RSC PureFood GMO and Authentication Kit (Promega) according to the protocol provided by the manufacturer. DNA concentration and quality were estimated using a NanoDrop 2000 (ThermoFisher Scientific, Warsaw, Poland). The DNA concentration was ranging from 116.2 to 1910 ng/µl (mean value 896 ± 369 ng/µl). The ratio of absorbance at 260 nm vs. 280 nm ranged from 1.87 to 2.20 (mean value 2.08 ± 0.06), whereas at 260 nm vs. 230 nm from 1.46 to 2.33 (mean value 2.06 ± 0.13).

### DArT-seq genotyping

DNA isolates from three biological replicates of every investigated genotype were diluted to 100 ng/µl and combined in equal aliquots to constitute bulked samples for DArTseq genotyping. The library preparation and the sequencing were conducted by Diversity Arrays Technology Pty Ltd. (University of Canberra, Bruce, Australia) using the Lupin DArTseq 1.0 protocol and high-density sequencing (2.5 mln reads). Obtained dataset included information about polymorphisms of the presence/absence variants (PAVs) of dominant markers (SilicoDArTs) and standard single nucleotide polymorphism (SNP) markers. To facilitate interpretation of the results, PAV markers were renamed to start with “M” letter whereas SNP markers to start with “N” letter.

### Sequence data Preparation

The raw DArT data for SNP and PAV markers was imported using dartR package^96^. Both files were subjected to four filtering steps, removing the following markers which: (a) were fully monomorphic in the given dataset (~ 231 000 markers), (b) showed the reproducibility rate below 97% (~ 600 markers) and (c) revealed the call rate by locus below 80% (~ 2000 markers). The output file was then submitted for the additional filtering using Microsoft Excel and following thresholds: maximum 20% of hetereozygotes, minimum 5% of minor allele frequency (MAF) and, again, maximum 20% of missing genotype calls. The reference alleles were randomly nominated based on the order of the sequence obtained. The sequences of DArT-seq markers with trimmed adaptor regions were aligned to the *L. luteus* genome (GenBank BioProject PRJEB74252, assembly GCA_964019355.1) using BLAST algorithm implemented in Geneious software^97,98^. The following parameters were applied: program blast n, word size 15, gap cost 5 (open) and 2 (extend), scoring 2 (match) and − 3 (mismatch), e-value threshold 1 × 10^− 5^, maximum number of hits 1. BLAST results were further filtered for the minimum pairwise identity of 90% and the minimum query coverage of 75%. *L. luteus* genome coordinates of retained blast hits were used for GWAS as well as for searching of candidate genes.

### Imputation of genotypes and population structure analysis

All marker data were transformed to 0, 1, 2 code, where 0 stands for the homozygote, 2 for the alternative allele homozygote, and 1 for the heterozygote. For data transformation, custom Python scripts were used. For missing data, imputation was performed using Beagle software version 4.1 ^99^ with its default settings. Duplicated loci with identical segregation were removed, leaving a single representative. That prepared data was filtered for minor allele frequency (MAF) with the threshold of 5.0%. Population structure analysis was performed using the snmf function from the LAE package^100^. This analysis estimated ancestral populations (K) in the range of 3 to 15, with 3000 replications and 5000 iterations for each K-value. The best run was determined using the cross-entropy criterion. For further analysis, population structure was visualized for the selected K = 6 using the pophelper package, facilitating clear representation of the Q matrix and the underlying population structure^101^.

### Genome-wide association mapping and visualization

GWAS was performed using two models: the Bayesian-information and Linkage-disequilibrium Iteratively Nested Keyway (BLINK) model^102^ and the Fixed and random model Circulating Probability Unification model (FarmCPU)^103^ implemented in the GAPIT R package^104^. In the analysis, we accounted for population structure (Q) through a Qmatrix and for relationships among individuals through a kinship (K) matrix^105^, both using the marker data. Based on the cross-entropy analysis, the Qmatrix of covariates with K = 6 was selected as the representative number of clusters for population structure in GWAS for each trait. The significance threshold for marker-trait associations (MTA) was set to *p* = 0.05 after applying the false discovery rate (FDR)^106^ correction. To control the False Discovery Rate (FDR) in genome-wide association analysis, the Benjamini-Hochberg (B-H) procedure was applied as implemented in the GAPIT package^104^, following the method described by Benjamini and Hochberg^107^.

To obtain genotype effect estimates across both years and vernalization treatments, we fitted a linear mixed model using the following structure:

$$\begin{aligned}Y&=\mu\:+{Genotype}^{(f)}+{Year}^{(f)}+{Vernalization}^{(f)}+{(Genotype\times\:Vernalization)}^{(f)}\\&+{(Genotype\times\:Year)}^{(r)}+{(Year:Pot:Row)}^{(r)}+{(Year:Pot:Column)}^{(r)}+\epsilon\end{aligned}$$

Where:

- **Genotype**: fixed effect (to extract unbiased BLUEs).
- **Year**: fixed effect (only two years observed).
- **Vernalization**: fixed effect (binary treatment: vernalized / non-vernalized).
- **Genotype × Vernalization**: fixed interaction term to model genotype-specific response to treatment.
- **Genotype × Year**: random interaction term to account for variation in genotype performance across years.
- **Row** and **Column** nested within **Pot** and **Year**: random effects to account for spatial heterogeneity in greenhouse conditions.
- **ε**: residual error.

The model was implemented in ASReml-R version 4.2 (VSN International, Hemel Hempstead, UK)^108^. Genotype was modeled as a fixed effect to extract BLUEs (Best Linear Unbiased Estimates), which were used as phenotypic input for genome-wide association analysis. This modeling strategy was selected to avoid double shrinkage, which can occur if the genotype is treated as random in both the phenotypic model and the GWAS. As discussed by Bernal-Vasquez et al.^109^, the use of BLUEs ensures unbiased estimation of genotype performance and is appropriate for two-stage GWAS pipelines. The mixed model also accounted for genotype × year interaction, treatment response (vernalization), and spatial heterogeneity in the greenhouse environment through nested row and column random effects.

Visualization of population structure, and violin plots was made in R software using packages ggplot2 ^110^, GAPIT^104^, and pophelper^111^. LD graphs were prepared using LDheatmap^112^, whereas Manhattan plots in the qqman package (https://cran.r-project.org/web/packages/qqman/index.html).

To find candidate genes, *L. luteus* genome regions carrying selected DArT-seq markers significantly associated with analyzed traits (LLUT_LOCUS sequences or ~ 1000 bp fragments if no annotation was found at particular marker locus) were aligned to the *L. albus* genome using BLAST online tool available on the genome website [https://www.whitelupin.fr/index.html]. Alignments with the lowest e-value were considered as significant and used for selection of candidate genes based on annotation provided for matching *L. albus* loci on the website genome browser^50,51^. Then, we conducted an analysis of the composition and arrangement of genes adjacent to the selected DArT markers, comparing gene order in the genome assemblies of *L. luteus* and *L. albus*. For genes lacking annotation or annotated as uncharacterized, we performed a BLAST search using NCBI and considered the annotation corresponding to the best match.

## Electronic supplementary material

Below is the link to the electronic supplementary material.

Supplementary Material 1

Supplementary Material 2

## Data Availability

The dataset generated and analyzed during the current study is available in the Zenodo repository (https://zenodo.org), at the link doi.org/10.5281/zenodo.15195286.
