# Supplementary material for: Molecular dissection of the genetic architecture of phenology underlying Lupinus hispanicus early flowering and adaptation to winter- or spring sowing
Source: Sci Rep. 2025 May 2;15:15324. doi: 10.1038/s41598-025-00096-1 (PMC12046050; doi:10.1038/s41598-025-00096-1)
Supplement: Supplementary file 2 — Supplementary Material 2 [file 41598_2025_96_MOESM2_ESM.pdf]

Title: Molecular dissection of the genetic architecture of phenology underlying *Lupinus hispanicus* early flowering and adaptation to winter- or spring sowing.

Authors: Wojciech Bielski, Anna Surma, Jolanta Belter, Bartosz Kozak, Michał Książkiewicz and Sandra Rychel-Bielska.

Scientific Reports

Supplementary Figures

Supplementary Figure S1. Values of the cross-entropy criterion for a number of clusters ranging from K3 to K15.

Calculations were performed for 173 *Lupinus hispanicus* accessions using 5959 SNP and 17769 PAV markers.

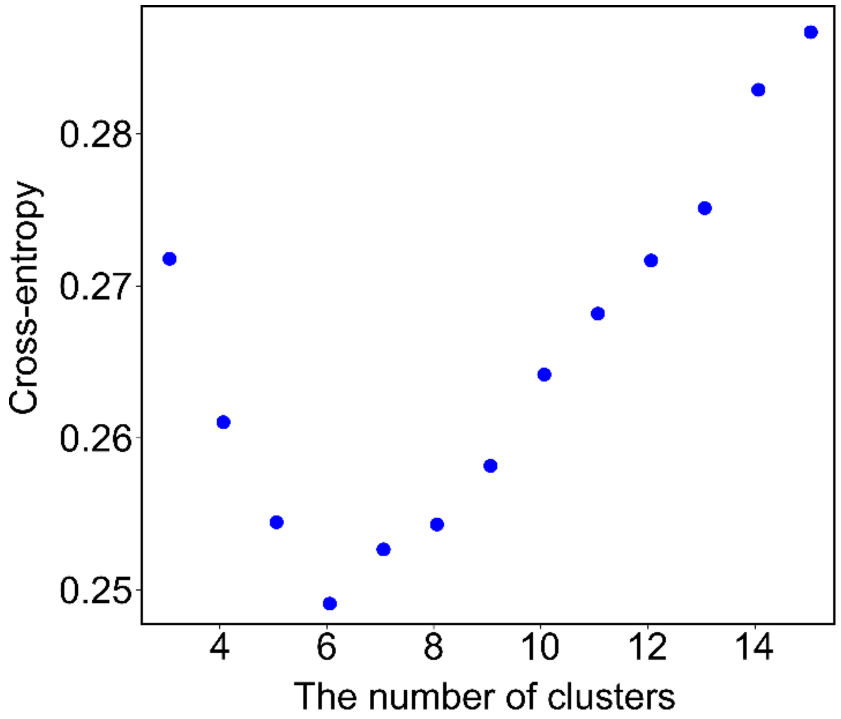

Supplementary Figure S2. The principal component analysis (PCA) showing the separation of major clusters of *Lupinus hispanicus* genotypes formed at K6.

A total of 5959 SNP and 17769 PAV markers were utilized for the analysis. The top figure illustrates PCA axis 1 versus axis 2, while the bottom figure shows PCA axis 2 versus axis 3.

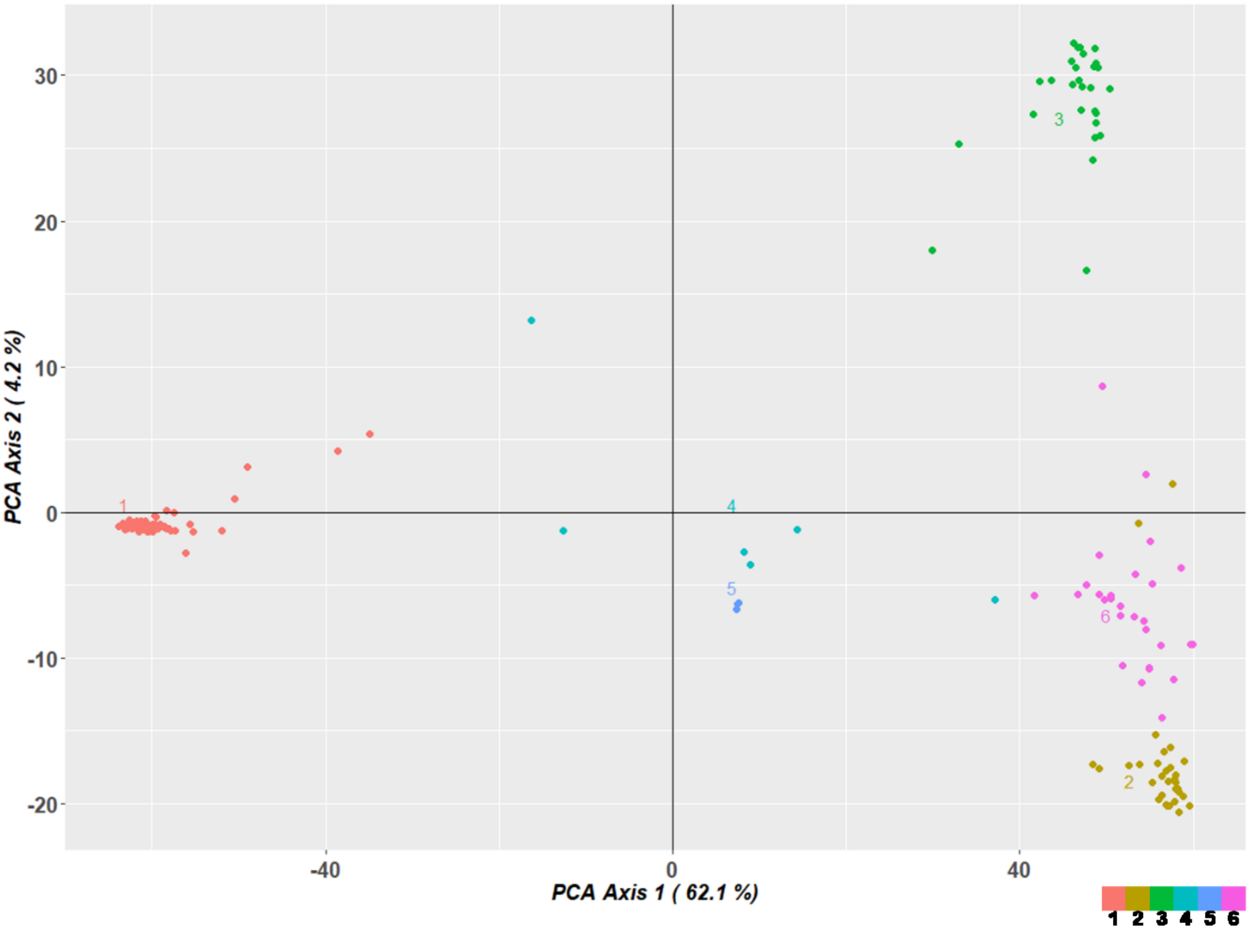

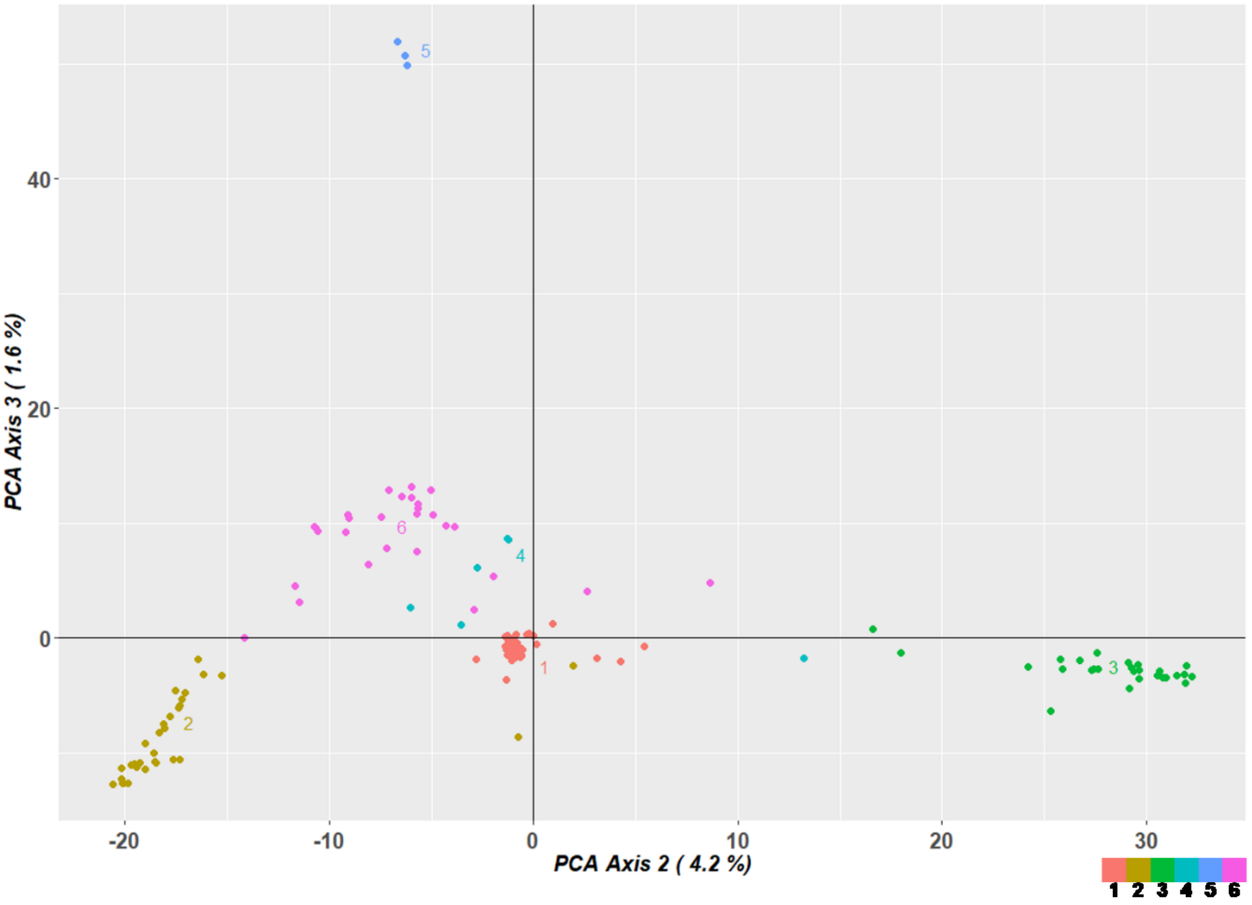

Supplementary Figure S3. Circular visualizations of Manhattan plots for genome-wide association study (GWAS) of *Lupinus hispanicus* germplasm panel.

Phenotypic observations were conducted in two separate glasshouse experiments in 2022 (Fig. 3a and Fig. 3b) and in 2023 (Fig. 3c and Fig. 3d.). The analyzed traits included the number of days which was recalculated as the cumulative number of growing degrees days (GDDs) for non-vernalized (N) and vernalized (V) plants from sowing until the floral bud emergence (traits NB_GDD_2022, NB_GDD_2023, VB_GDD_2022, and VB_GDD_2023), the start of flowering (NF_GDD_2022, NF_GDD_2023, VF_GDD_2022 and VF_GDD_2023) and pod maturity (NP_GDD_2022, NP_GDD_2023, VP_GDD_2022 and VP_GDD_2023). Two algorithms, BLINK (Fig. 3a and 3c) and FarmCPU (Fig. 3b and Fig. 3d), were used for the GWAS calculations.

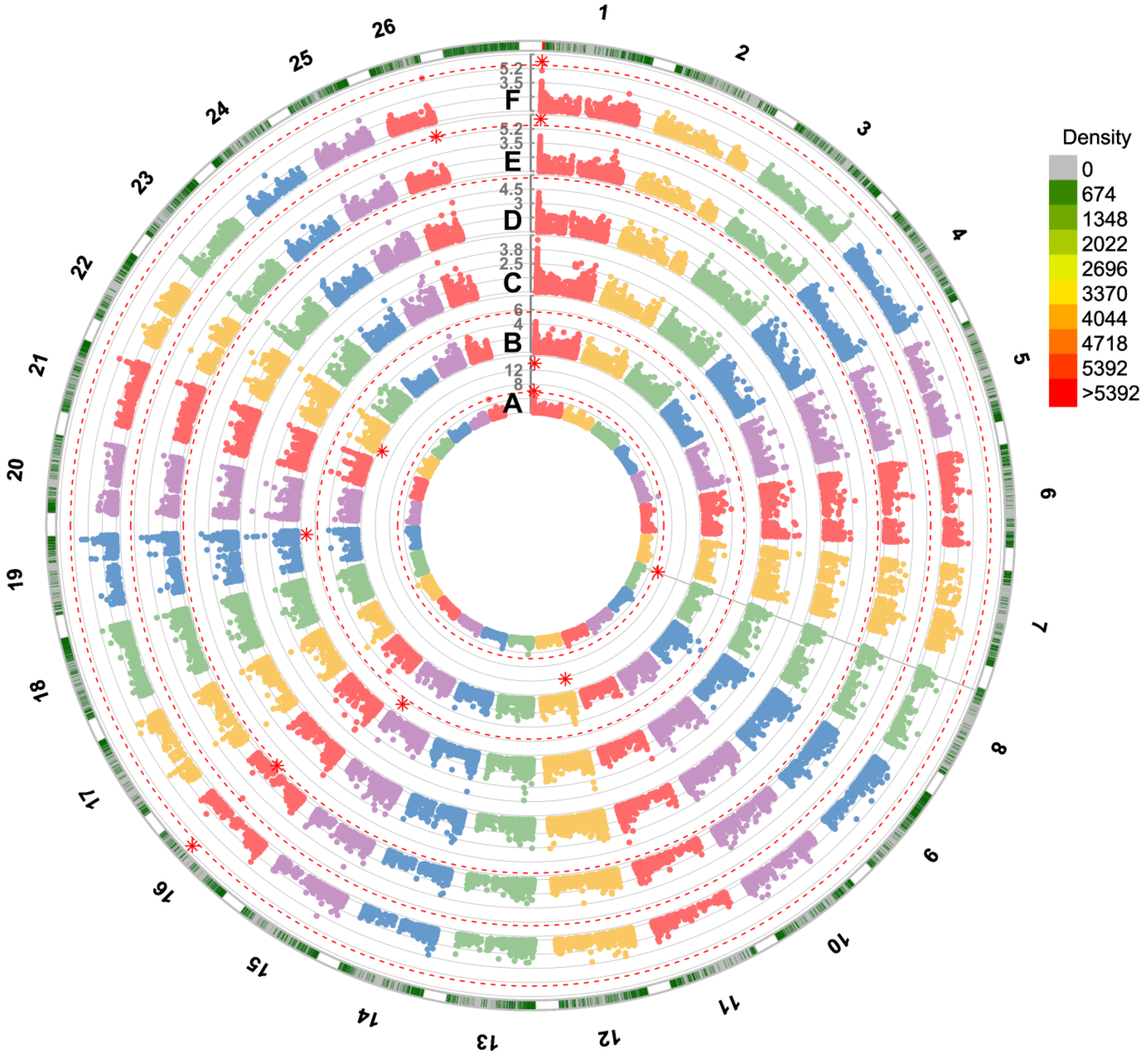

Figure S3a. Circular visualizations of Manhattan plots for BLINK-based GWAS of GDDs for observations performed in 2022. Six different traits are visualized: A – GDDs of non-vernalized plants until the floral bud emergence; B – GDDs of non-vernalized plants until the start of flowering; C – GDDs of non-vernalized plants until pod maturity; D – GDDs of vernalized plants until the floral bud emergence; E – GDDs of vernalized plants until the start of flowering; F – GDDs of vernalized plants until pod maturity. Asterisks highlighted FDR-corrected significantly associated markers.

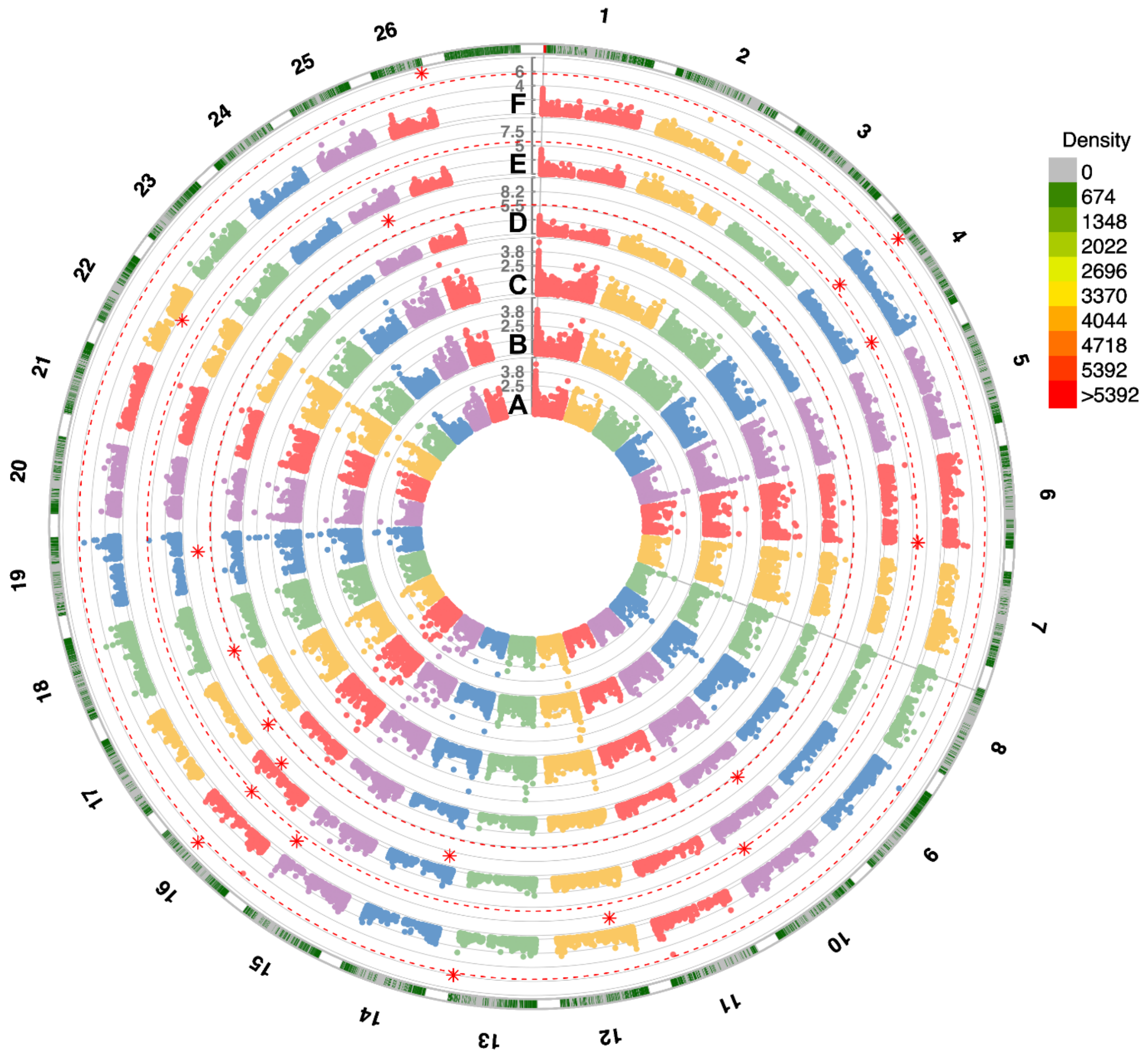

Figure S3b. Circular visualizations of Manhattan plots for FarmCPU-based GWAS of GDDs for observations performed in 2022. Six different traits are visualized: A – GDDs of non-vernalized plants until the floral bud emergence; B – GDDs of non-vernalized plants until the start of flowering; C – GDDs of non-vernalized plants until pod maturity; D – GDDs of vernalized plants until the floral bud emergence; E – GDDs of vernalized plants until the start of flowering; F – GDDs of vernalized plants until pod maturity. Asterisks highlighted FDR-corrected significantly associated markers.

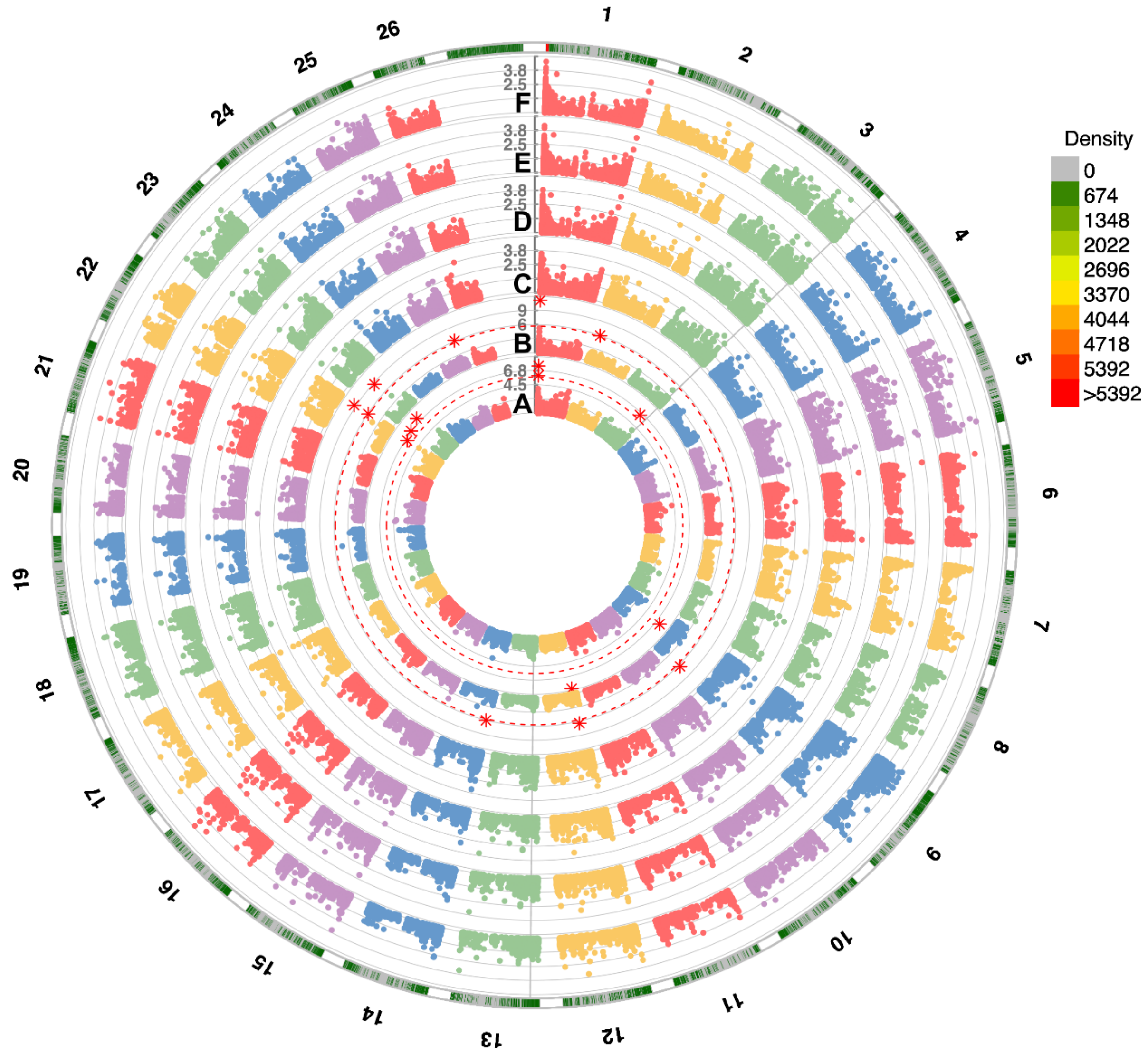

Figure S3c. Circular visualizations of Manhattan plots for BLINK-based GWAS of GDDs for observations performed in 2023. Six different traits are visualized: A – GDDs of non-vernalized plants until the floral bud emergence; B – GDDs of non-vernalized plants until the start of flowering; C – GDDs of non-vernalized plants until pod maturity; D – GDDs of vernalized plants until the floral bud emergence; E – GDDs of vernalized plants until the start of flowering; F – GDDs of vernalized plants until pod maturity. Asterisks highlighted FDR-corrected significantly associated markers.

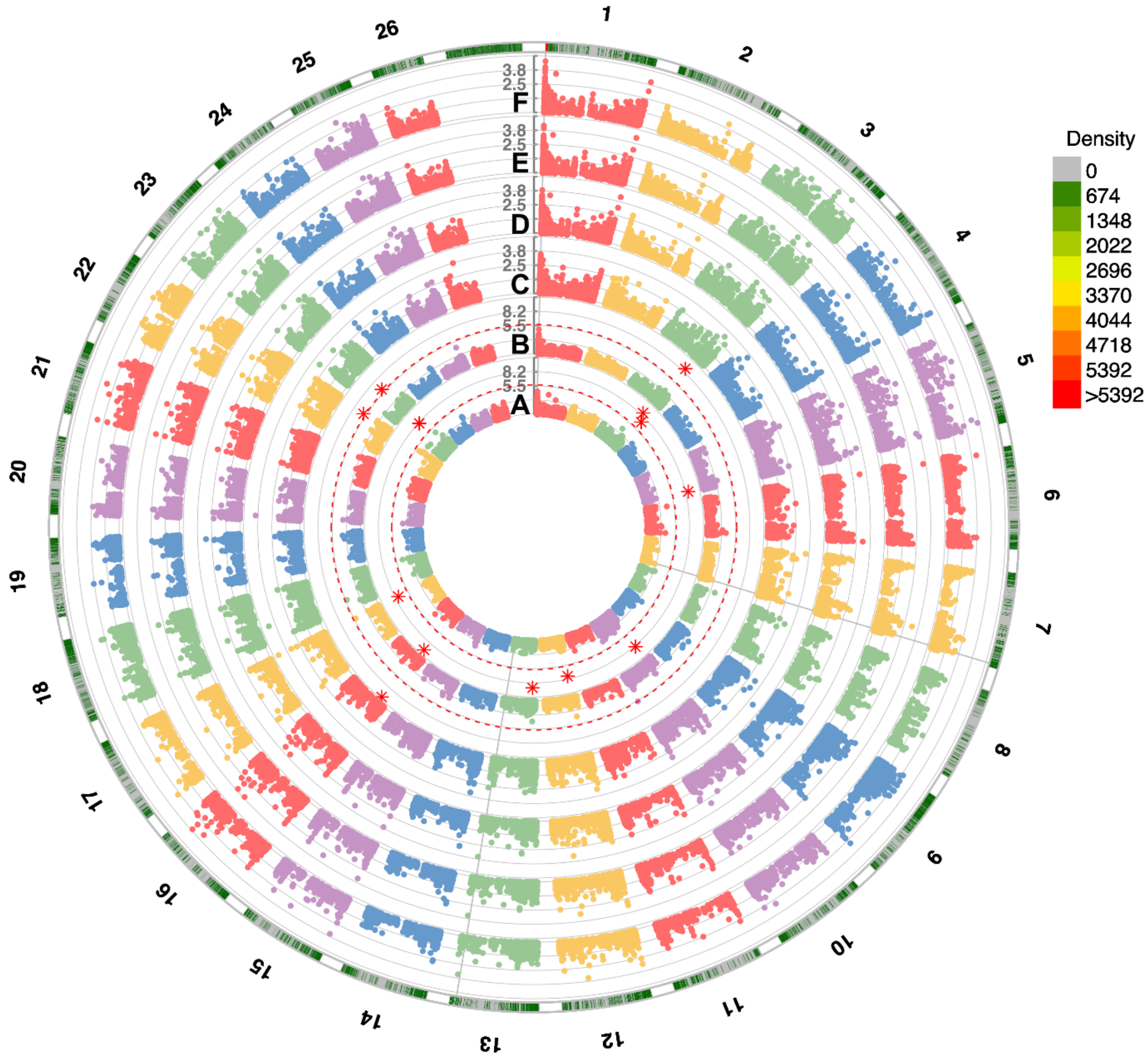

Figure S3d. Circular visualizations of Manhattan plots for FarmCPU-based GWAS of GDDs for observations performed in 2023. Six different traits are visualized: A – GDDs of non-vernalized plants until the floral bud emergence; B – GDDs of non-vernalized plants until the start of flowering; C – GDDs of non-vernalized plants until pod maturity; D – GDDs of vernalized plants until the floral bud emergence; E – GDDs of vernalized plants until the start of flowering; F – GDDs of vernalized plants until pod maturity. Asterisks highlighted FDR-corrected significantly associated markers.
